# Superior visible light hydrogen evolution of Janus bilayer junctions via atomic-level charge flow steering

**DOI:** 10.1038/ncomms11480

**Published:** 2016-05-09

**Authors:** Jie Li, Guangming Zhan, Ying Yu, Lizhi Zhang

**Affiliations:** 1 Key Laboratory of Pesticide and Chemical Biology of Ministry of Education, Institute of Environmental Chemistry, College of Chemistry, Central China Normal University, Wuhan 430079, China; 2 Institute of Nanoscience and Nanotechnology, College of Physical Science and Technology, Central China Normal University, Wuhan 430079, China

## Abstract

Although photocatalytic hydrogen evolution (PHE) is ideal for solar-to-fuel conversion, it remains challenging to construct a highly efficient PHE system by steering the charge flow in a precise manner. Here we tackle this challenge by assembling 1T MoS_2_ monolayers selectively and chemically onto (Bi_12_O_17_) end-faces of Bi_12_O_17_Cl_2_ monolayers to craft two-dimensional (2D) Janus (Cl_2_)-(Bi_12_O_17_)-(MoS_2_) bilayer junctions, a new 2D motif different from van der Waals heterostructure. Electrons and holes from visible light-irradiated Bi_12_O_17_Cl_2_ are directionally separated by the internal electric field to (Bi_12_O_17_) and (Cl_2_) end-faces, respectively. The separated electrons can further migrate to MoS_2_ via Bi–S bonds formed between (Bi_12_O_17_) and MoS_2_ monolayers. This atomic-level directional charge separation endows the Janus bilayers with ultralong carrier lifetime of 3,446 ns and hence a superior visible-light PHE rate of 33 mmol h^−1^ g^−1^. Our delineated Janus bilayer junctions on the basis of the oriented assembly of monolayers presents a new design concept to effectively steer the charge flow for PHE.

## Abstract (web summary)

The efficiency of photocatalytic hydrogen evolution is impeded by the inability of semiconductors to precisely steer the charge flow. Here, the authors address this issue by designing conceptually new Janus bilayer junctions.

## Graphical abstract

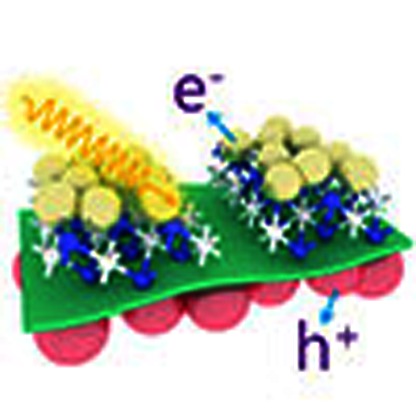

Photocatalytic hydrogen evolution (PHE) from water via solar energy and semiconductor photocatalysts provides the prospect of replacing fossil fuels with carbon-free and sustainable hydrogen energy^1^. Yet so far, its realization is still a mirage because PHE efficiency is thwarted to a large extent by the undesirable electron–hole recombination arisen from the random charge flow after the electron–hole separation^2^. The solution of this challenging issue requires our understanding and tuning of charge flow from the microcosmic perspective, preferably at the atomic level, which is still beyond the current state-of-the-art advances on the charge flow steering.

Recent flourishes in graphene studies are provoking tremendous interest in exfoliation-based fabrication of 2D single-layered nanosheets^3^. These advances breathe new life into PHE, as the as-exfoliated 2D monolayers with atomically thin thicknesses offer access to the atomic-level understanding and steering of charge flow^4^,^5^,^6^,^7^. Such monolayers enable atomic-level control over architectures and electronic structures, thus manipulating the charge flow from the electron–hole separation sites to their surface in a desired route^8^,^9^. However, individual semiconductor monolayers still encounter substantial charge recombination for PHE, because they lack sufficient hydrogen-evolving sites to manipulate the fates of these surface-reaching electrons^10^,^11^. To address this issue and also realize the charge flow at the atomic level, it is highly desirable to load monolayers with abundant hydrogen-evolving sites, such as single-layered metallic transition metal dichalcogenides, selectively on electron-accumulated sites of semiconductor monolayers to extract electrons. Such assembly design may steer the separation, transportation and consumption of charge all at the atomic level, but at present remains unexplored.

In this study, we report on the synthesis of novel bilayer junctions by assembling metallic MoS_2_ monolayers selectively and chemically on oxygen-deficient (Bi_12_O_17_) end-faces of Bi_12_O_17_Cl_2_ semiconductor monolayers via oxygen-vacancy (OV) chemistry. With the resultant 2D Janus motif of (Cl_2_)- (Bi_12_O_17_)- (MoS_2_), we can successfully steer the charge separation, transportation and consumption all at the atomic level to construct a highly active PHE system. In such a system, electrons originating from visible light-irradiated Bi_12_O_17_Cl_2_ are first driven by the internal electric field (IEF) between (Cl_2_) and (Bi_12_O_17_) layers to (Bi_12_O_17_) end-faces, and further transferred via Bi–S bonds formed between Bi_12_O_17_Cl_2_ and MoS_2_ to MoS_2_ for the final catalytic hydrogen evolution, while IEF also drives holes to (Cl_2_) end-faces for the oxidation of organic scavengers.

## Results

### Assembly and characterization of the Janus bilayer junctions

**Figure 1:**
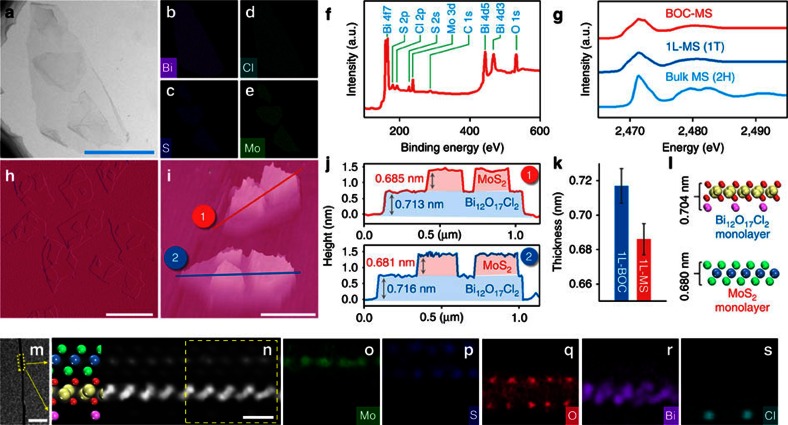
Characterizations of the Janus bilayer junctions assembled by Bi_12_O_17_Cl_2_ and MoS_2_ monolayers (BOC-MS). (**a**) Top-view TEM image, (**b**–**e**) elemental mapping images, (**f**) XPS spectra, (**h**,**i**) AFM images, side-view TEM image (**m**) and atomic-resolution HAADF-STEM image (**n**), and (**o**–**s**) the corresponding EELS elemental maps of BOC-MS. Scale bar, 500 nm, 1 μm, 500 nm, 10 nm and 5 Å, in **a**,**h**,**i**,**m**,**n**, respectively. (**g**) S K-edge XANES spectra of BOC-MS, 1L-MS and bulk MS. (**j**) Height profiles along the lines in **i**. (**k**) Comparison of the average thicknesses of 1L-BOC and 1L-MS in BOC-MS. The error bars in **k** represent the s.d. of over 100 independent AFM measurements. (**l**) The theoretical thicknesses of MoS_2_ and Bi_12_O_17_Cl_2_ monolayers.

Small-sized MoS_2_ monolayers (1L-MS) enriched with metallic phase and large-sized oxygen-deficient Bi_12_O_17_Cl_2_ semiconductor monolayers (1L-BOC) were obtained by exfoliating their bulk layered mother-crystals via the well-established lithium-intercalation-based liquid exfoliation (Supplementary Figs 1–4 and Supplementary Note 1–4)^12^,^13^. The experimentally measured thickness of ∼0.71 nm in 1L-BOC well-agreed with the theoretical one (0.2c=0.704 nm) of Bi_12_O_17_Cl_2_ monolayer (Supplementary Figs 2 and 3 and Supplementary Note 2 and 3), as Bi_12_O_17_Cl_2_ had a c=35.2 Å and a unit cell consisting of five Bi_12_O_17_Cl_2_ monolayers, similar with the reported Bi_2_WO_6_ (ref. 9), which had a c=16.427 Å, a unit cell consisting of two Bi_2_WO_6_ monolayers, but a monolayer thickness of 0.5c≈0.8 nm as experimentally measured. The use of OV chemistry to craft the aforementioned Janus bilayer junctions (BOC-MS) was inspired by the intriguing capability of OV-deficient metal oxides to induce the selective deposition of metals on their OV sites^14^. Enlightened by this methodology, we assembled the above two monolayers with a facile hydrothermal processing, where OVs induced the oriented anchoring of 1L-MS on (Bi_12_O_17_) end-faces of Bi_12_O_17_Cl_2_ to form 2D Janus bilayer junctions of (Cl_2_)-(Bi_12_O_17_)-(MoS_2_). The key to this OV-oriented assembly lies in the metallic characteristic of 1L-MS and the asymmetric structure of 1L-BOC composed of only (Cl_2_) layers and oxygen-deficient (Bi_12_O_17_) layers. Transmission electron microscopy (TEM) image (Fig. 1a) of BOC-MS revealed a 2D heterostructured bilayer composed of many small nanosheets tightly wrapping on a large nanosheet, while the observed transparent nature indicated their ultrathin structures. Elemental mapping images (Fig. 1b–e) coupling with X-ray photoelectron spectroscopy (XPS) (Fig. 1f) and X-ray diffraction pattern (Supplementary Fig. 5 and Supplementary Note 5) identified the small and large nanosheets as MoS_2_ and Bi_12_O_17_Cl_2_, respectively. S K-edge X-ray absorption near-edge structure spectra (XANES) (Fig. 1g) revealed that the MoS_2_ monolayers in the bilayer showed a distorted 1T metallic phase^15^. 3D topographic atomic force microscopy (AFM) images (Fig. 1h,i) and their corresponding height profiles (Fig. 1j,k) demonstrated that the average thickness values of the small-sized and large-sized nanosheets were 0.686 and 0.717 nm, respectively, well-matching with the theoretical ones of the MoS_2_ and Bi_12_O_17_Cl_2_ monolayers (Fig. 1l, Supplementary Figs 2–4 and Supplementary Note 2–4), which further evidenced the assembly of MoS_2_ on Bi_12_O_17_Cl_2_. More interestingly, we found that all the MoS_2_ sheets were anchored on the same surface in Bi_12_O_17_Cl_2_, which was further evidenced by their side-view TEM image (Fig. 1m). These observations clearly demonstrate the occurrence of an oriented assembly. As 1L-BOC has an asymmetric structure consisting of (Cl_2_) and oxygen-deficient (Bi_12_O_17_) end-faces and their assembly might be initiated by OVs of (Bi_12_O_17_) end-faces, we could thus deduce that 1L-MS were anchored selectively on the (Bi_12_O_17_) end-faces of 1L-BOC. The aberration-corrected high-angular annular dark field scanning TEM (HAADF-STEM) image (Fig. 1n) and energy loss spectroscopy (EELS) elemental maps (Fig. 1o–s) of their cross-sectional atomic structures provided direct, atomic-resolution evidences that this oriented assembly resulted in 2D Janus bilayer junctions of (Cl_2_)-(Bi_12_O_17_)-(MoS_2_). Meanwhile, we also found that the amount of MoS_2_ loaded on Bi_12_O_17_Cl_2_ decreased with OVs quenching, and the assembly behaviour annihilated when OVs were completely removed (Supplementary Figs 5–7, Supplementary Table 1 and Supplementary Note 6 and 7), confirming that the oriented assembly of 2D Janus (Cl_2_)-(Bi_12_O_17_)-(MoS_2_) bilayer junctions was driven by the OVs on the (Bi_12_O_17_) end-faces of 1L-BOC.

### Visible light PHE

**Figure 2:**
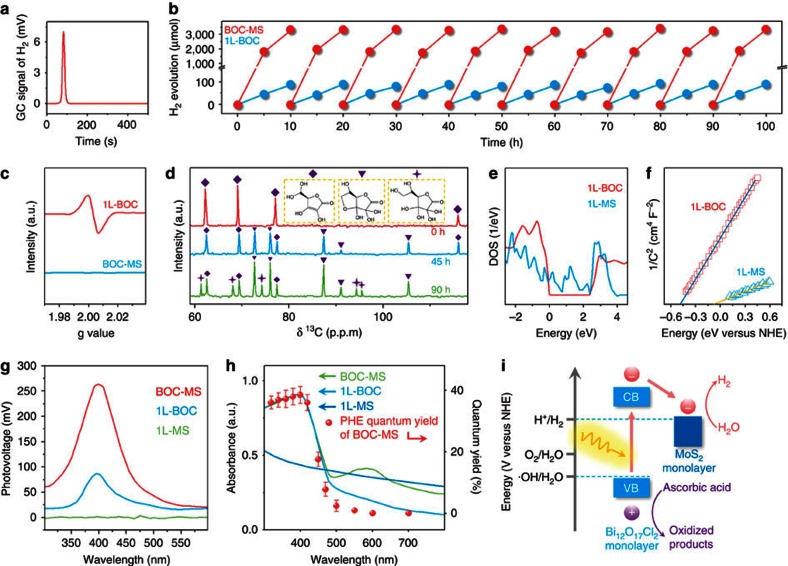
Evaluation of visible light PHE performances of BOC-MS. (**a**) A typical GC signal of H_2_ formed from BOC-MS involved PHE system. (**b**) Cycling tests of PHE over BOC-MS and 1L-BOC. Reaction conditions: 10 mg catalysts; 0.3 mol l^−1^ ascorbic acid; visible light (*λ*>420 nm). The reaction cell was evacuated each 10 h without renewing ascorbic acid solution. (**c**) EPR spectra of 1L-BOC and BOC-MS. (**d**) 13C NMR spectra of the solutions containing BOC-MS and ascorbic acid after 0, 45 and 90 h of PHE experiments. (**e**) DOS and (**f**) Mott–Schottky plots of 1L-BOC and 1L-MS. (**g**) Surface photovoltage spectroscopy of 1L-BOC, 1L-MS and BOC-MS. (**h**) Ultraviolet–visible diffuse reflectance spectrum of BOC-MS, 1L-BOC and 1L-MS, and PHE quantum yields of BOC-MS plotted as a function of wavelength of the incident light. (**i**) Band alignments in 1L-BOC and 1L-MS.

The successful design of BOC-MS featuring Janus (Cl_2_)-(Bi_12_O_17_)-(MoS_2_) bilayer junctions allowed us to first explore their PHE activity under visible light (λ>420 nm). Visible light irradiation of the solution containing BOC-MS and ascorbic acid (organic scavengers) gave a typical gas chromatograph (GC) signal of H_2_ at the retention time of 82 s (Fig. 2a), consistent with the previously reported results^16^. At the optimal MoS_2_ loading (10 wt%, Supplementary Fig. 8a) and ascorbic acid concentration (0.3 mol l^−1^, Supplementary Fig. 8b), BOC-MS delivered a PHE rate of 33 mmol h^−1^ g^−1^ (Fig. 2b) and a quantum yield of around 36% at 420 nm (Fig. 2h), representing the currently achieved state-of-the-art PHE activity among all the MoS_2_-based, monolayer-based and bismuth oxyhalide-based PHE systems (Supplementary Tables 2–4). This activity could last over 100 h without significant decay and the bilayer structures were very stable (Supplementary Fig. 9 and Supplementary Note 8), indicating BOC-MS were highly active and robust for visible-light PHE. The absence of OVs signal in BOC-MS ruled out the possibility that its superior PHE activity was arisen from the OVs (Fig. 2c). During PHE, dehydroascorbic acid and its hydrated compound as the oxidized products of ascorbic acid were detected (Fig. 2d)^17^, indicating that ascorbic acid acted as an efficient hole scavenger to suppress the electron and hole recombination. Even using ascorbic acid as the hole scavenger and having surface OVs, 1L-BOC only exhibited a PHE rate of 0.86 mmol h^−1^ g^−1^, 1/38 that of BOC-MS.

This giant PHE activity enhancement by the introduction of 1L-MS ignited our interest in unravelling how 1L-MS assisted 1L-BOC to precisely steer charge flow across the assembled Janus bilayer junctions. Density functional theory calculations of 1L-MS showed its density of states resided across the Fermi level (Fig. 2e), revealing its metallic characteristic^3^, which indicated that 1L-MS favoured the electron collection. Mott–Schottky plots showed that 1L-BOC had the much more negative conduction band potential than 1L-MS (Fig. 2f and Supplementary Fig. 10), suggesting that the electron transfer from 1L-BOC to 1L-MS was thermodynamically more favourable. As 1L-MS showed no photoresponse and its assembly onto 1L-BOC could enhance the photoresponse (Fig. 2g), we could conclude that 1L-BOC in the photoexcited BOC-MS solely offered electrons and these electrons were further transferred from 1L-BOC to 1L-MS for PHE (Fig. 2i). This conclusion was confirmed by the observation that the irradiation wavelength-dependent quantum yield of PHE matched well with the photoabsorption edge of 1L-BOC, not with those of 1L-MS or BOC-MS (Fig. 2h and Supplementary Fig. 11).

### IEF-directed charge flow within Bi_12_O_17_Cl_2_ monolayers

**Figure 3:**
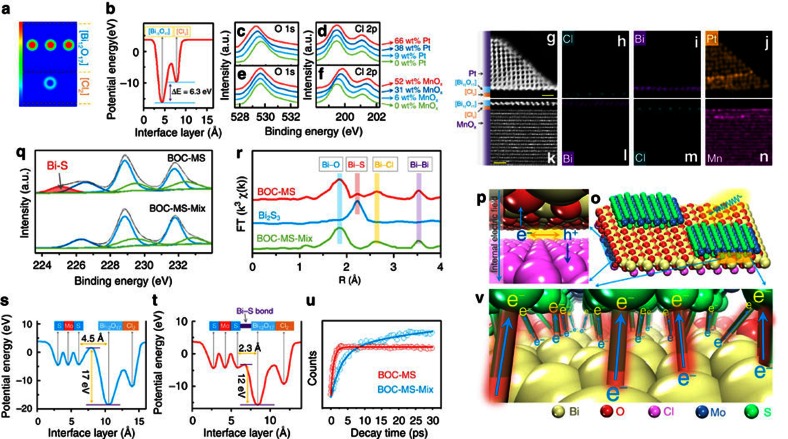
Clarification of the charge flow within visible light-irradiated BOC-MS. (**a**) Charge density contour plots and (**b**) electrostatic potential viewed from the {110} facet of Bi_12_O_17_Cl_2_. XPS spectra of 1L-BOC photo-deposited with different amounts of Pt (**c**,**d**) and MnO_x_ (**e**,**f**), demonstrating the interactions between Pt (or MnO_x_) and atoms on deposition sites. (**g**,**k**) The side-view atomic-resolution HAADF-STEM images, and (**h**–**j**,**l**–**n**) the corresponding EELS elemental maps of 1L-BOC photo-deposited with Pt and MnO_x_. Scale bar, 10 Å in **g**,**k**. Comparison of XPS spectra (**q**) and Bi L-edge EXAFS (**r**) of BOC-MS and BOC-MS-Mix, identifying the formation of Bi–S bonds at the interfaces between Bi_12_O_17_Cl_2_ and MoS_2_ in the Janus bilayer. Comparison of the electrostatic potentials of (Cl_2_)-(Bi_12_O_17_)-(MoS_2_) with (**s**) and without (**t**) Bi–S bonds. (**u**) Comparison of the build-up dynamics of electrons' excited-state absorption (ESA) signals (pumped at 400 nm and probed at 650 nm) of BOC-MS and BOC-MS-Mix, highlighting the crucial role of Bi–S bonds in steering charge flow from 1L-BOC to 1L-MS in BOC-MS. Schematic illustration of the crystal structure of BOC-MS (**o**) and of the charge flow processes within BOC-MS, including the electron–hole separation within 1L-BOC (**p**) and the interfacial electron transfer from 1L-BOC to 1L-MS along the Bi–S bonds (**v**).

To clarify these detailed charge flow processes, we first examined the charge flow in 1L-BOC. Charge density contour plots viewed from the {110} facet of Bi_12_O_17_Cl_2_ (Fig. 3a) showed that the charge density surrounding (Bi_12_O_17_)^2+^ layer was higher than that of (Cl_2_)^2−^ layer and their electrostatic potential differences (ΔE) was calculated to be 6.3 eV (Fig. 3b). This non-uniform charge distribution between (Bi_12_O_17_)^2+^ and (Cl_2_)^2−^ layers in Bi_12_O_17_Cl_2_ nanosheets would polarize the related atoms and orbitals to form a permanent IEF along [001] orientation perpendicular to the (Bi_12_O_17_)^2+^ and (Cl_2_)^2−^ layers^18^,^19^,^20^. Using the Kanata's model (Supplementary Methods), which has been used to successfully identify the dependence of IEF intensity of Bi_3_O_4_Cl on its {001} facet exposure percentages^20^, we found that monolayer engineering could enhance the IEF magnitude of Bi_12_O_17_Cl_2_ by sixfolds (Supplementary Fig. 12). As 1L-BOC possessed an asymmetric structure composed of only (Cl_2_)^2−^ and (Bi_12_O_17_)^2+^ layers (Supplementary Fig. 3f), we proposed that its IEF would drive electrons and holes to (Bi_12_O_17_)^2+^ and (Cl_2_)^2−^ end-faces, respectively. To confirm this atomic-level charge separation, we thus deposited Pt and MnO_x_ on 1L-BOC with H_2_PtCl_6_ and Mn(NO_3_)_2_ as the respective precursors under visible light. We determined their deposition sites through the interaction between Pt (or MnO_x_) and the atoms at deposition sites via XPS. The Pt deposition resulted in a shift of O 1 s peak position toward lower binding energy without affecting Cl 2p peak position (Fig. 3c,d), indicating the interactions between Pt and O on (Bi_12_O_17_) end-faces. As for the MnO_x_ deposition, only the Cl 2p peak position shift toward higher binding energy was observed (Fig. 3e,f), indicating the interactions between MnO_x_ and (Cl_2_) end-faces. When the amounts of photo-deposited Pt and MnO_x_ increased, their size distributions did not change (Supplementary Figs 13 and 14) and the peak shifts became more striking, strongly confirming the photo-depositions of Pt on (Bi_12_O_17_) end-faces and MnO_x_ on (Cl_2_) end-faces, respectively. These selective photo-depositions were further validated by Raman spectra (Supplementary Fig. 15), which revealed that the intensities of Bi–O and Cl–Cl stretching bands were suppressed by Pt and MnO_x_ depositions, respectively. The atomic-resolution HAADF-STEM images (Fig. 3g,k) and EELS elemental maps (Fig. 3h–j,l–n) of the cross-sectional atomic structures of 1L-BOC photo-deposited with Pt and MnO_x_ provided direct and visual evidences that Pt was photo-deposited on the (Bi_12_O_17_) end-faces and MnO_x_ on the (Cl_2_) end-faces. As Pt and MnO_x_ depositions were induced by electron reduction and hole oxidation, respectively^21^, we could therefore conclude that, in 1L-BOC, IEF drove electrons to (Bi_12_O_17_) end-faces and holes to (Cl_2_) end-faces (Fig. 3o,p), achieving the atomic-level steering of charge separation. Although the 1L-BOC were rich in surface OVs, the selective deposition of Pt was independent on the OVs on the (Bi_12_O_17_) end-surfaces, as evidenced by the oriented anchoring of Pt on OV-free (Bi_12_O_17_) end-surfaces (Supplementary Fig. 16). This phenomenon suggested the above charge separation was not induced by OVs, but further verified the role of IEF in steering the atomic-level charge separation. As a result of this directional charge separation, 1L-BOC afforded a carrier lifetime of 136 ns and a PHE rate of 0.86 mmol h^−1^ g^−1^ (Supplementary Fig. 17), 15 and 27 times those of bulk Bi_12_O_17_Cl_2_, respectively. This is because, for bulk Bi_12_O_17_Cl_2_ of numerous (Bi_12_O_17_) and (Cl_2_) layers, the electrons and holes separated by IEF would recombine on the sites between the two adjacent Bi_12_O_17_Cl_2_ monolayers, leading to the short-lived carrier lifetime and hence the low PHE activity.

### Interfacial atomic-level charge flow along Bi–S bonds

After clarifying the photogenerated electrons flow to (Bi_12_O_17_) end-faces driven by IEF, we further checked the interfacial properties of the Janus bilayer junctions to unravel how the electrons transferred from (Bi_12_O_17_) end-faces of 1L-BOC to 1L-MS. XPS of BOC-MS showed a Bi–S bond peak at 225 eV (Fig. 3q)^22^, but absent in those of 1L-MS and the physical mixtures of 1L-MS and 1L-BOC (BOC-MS-Mix), and this peak became weaker in the bilayers assembled by 1L-MS and Bi_12_O_17_Cl_2_ monolayers with less OVs (Supplementary Fig. 18a). Bi L-edge extended X-ray absorption fine structure spectroscopy (Fig. 3r) also validated the existence of Bi–S bonds between the interfaces of 1L-MS and 1L-BOC in BOC-MS^22^,^23^. This interfacial Bi–S bonds could be further evidenced by the Raman spectra (Supplementary Fig. 19e) and diffuse reflectance Fourier transform infrared curves (Supplementary Fig. 19f and Supplementary Note 9). Furthermore, because of the interfacial chemical bonding via the Bi–S bonds, MoS_2_ and Bi_12_O_17_Cl_2_ monolayers in the Janus bilayers could enhance their respective thermal decomposition temperatures by 104 and 87 °C (Supplementary Fig. 19g,h,j). In contrast, no obvious improvement was observed in the thermal decomposition temperature of BOC-MS-Mix without the interfacial chemical Bi–S bonds (Supplementary Fig. 19i). Therefore, all these characterizations provided strong evidences for the existence of Bi–S bonds in the interface between Bi_12_O_17_Cl_2_ and MoS_2_ monolayers. The formation of Bi–S bonds and the dependence of their XPS peak intensity on the OV amounts of 1L-BOC indicated that OV chemistry could selectively assemble 1L-MS on (Bi_12_O_17_) end-faces of 1L-BOC, and also chemically solidify the interfaces between 1L-BOC and 1L-MS with the resultant Bi–S bonds. It is worth noting that the Janus bilayer junctions of (Cl_2_)-(Bi_12_O_17_)-(MoS_2_) that we designed and demonstrated here is a new motif for 2D material family, because it is precisely assembled and has chemical bonds as the main linkers between the constituent monolayers. The emergence of Bi–S bonds could be attributed to the OV-mediated exposure of the coordinately unsaturated Bi atoms, which interacted with the surface-terminated S atoms of 1L-MS to form Bi–S bonds during hydrothermal processing. Different from Van der Waals heterostructures^24^, such as BOC-MS-Mix, which lacked the electron shuttle pathway between two adjacent component layers, these chemical junctions may utilize the Bi–S bonds as the interfacial charge flow highway to enable more facile electron transfer from (Bi_12_O_17_) layers to 1L-MS. To verify this interfacial electron transfer, we calculated the electrostatic potentials of (Cl_2_)-(Bi_12_O_17_)-(MoS_2_) with and without Bi–S bonds. When 1L-BOC and 1L-MS interacted with Van der Waals force, the electron transfer from 1L-BOC to 1L-MS needed overcome an energy barrier of 17 eV and the transfer distance was 4.5 Å (Fig. 3s). The energy barrier and transfer distance could be decreased by 5 eV and 2.2 Å, respectively, by the introduction of the Bi–S bonds (Fig. 3t), indicating that the electron transfer along Bi–S bond was energetically and spatially more favourable. To support this theoretical calculation we used femtosecond-resolved transient absorption (TA) spectroscopy to decode the build-up dynamics of electrons' excited-state absorption signals. The TA curves of both BOC-MS and BOC-MS-Mix gave three lifetime components (Fig. 3u and Supplementary Fig. 20), among which τ_1_, τ_2_ and τ_3_, with relative intensities *I*_*1*_, *I*_*2*_ and *I*_*3*_, are rationally assigned to the electron migration along the Bi–S bonds, the electron trapping by surface states and the electron hopping within Van der Waals heterostructures^25^,^26^. It was found that *I*_*1*_ increased ca. 17-fold from 5.23% for BOC-MS-Mix to 86.59% for BOC-MS, and its magnitude highly depended on the amounts of Bi–S bonds, confirming the electron transport from 1L-BOC to 1L-MS via the Bi–S bonds within BOC-MS (Fig. 3v). This interfacial electron transfer was ultrafast with a rate of up to 5.8 × 10^−11^ s^−1^, as calculated by (τ_BOC-MS_)^−1^−(τ_BOC-MS-Mix_)^−1^, where τ_BOC-MS_ and τ_BOC-MS-Mix_ are the average TA lifetimes for BOC-MS and BOC-MS-Mix, respectively. As a result, the electron flow from 1L-BOC to 1L-MS required only 0.68 ps (τ_1_ for BOC-MS), which was inferior to the time scale (3–20 ps) required for the surface charge recombination^1^,^2^,^27^. This comparison indicated that, in photoexcited BOC-MS, the separated electrons of 1L-BOC preferred to migrate along the Bi–S bonds to 1L-MS, rather than recombine with the surface holes. Besides the aforementioned atomic-level steering of the first step charge flow from the electron–hole separation sites to the surface of 1L-BOC by monolayer engineering, such an ultrafast, efficient and selective extracting of electrons offered by the Bi–S bonds maintained the second-step charge flow from the surface of 1L-BOC to hydrogen-evolving sites still at the atomic level because of the MoS_2_ monolayer nature. The combination of atomic-level charge separation steering in 1L-BOC and atomic-level charge flow control between the interfaces of 1L-BOC and 1L-MS thus endowed BOC-MS with an ultralong carrier lifetime up to 3,446 ns (Supplementary Fig. 18b), far exceeding those of 1L-BOC (136 ns), and BOC-MS-Mix (165 ns). More importantly, by means of this atomic-level interfacial design for the sufficient electrons extracting from (Bi_12_O_17_) end-faces, BOC-MS exhibited a PHE rate 20 times higher than that of BOC-MS-Mix (Supplementary Fig. 18c).

### Atomic-level charge flow across the Janus bilayer junctions

**Figure 4:**
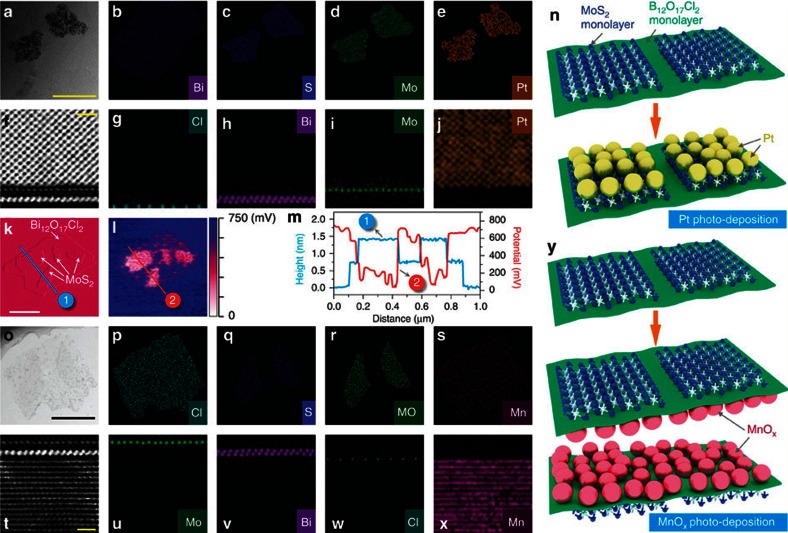
Imaging of the destinations of electrons and holes in visible light-irradiated BOC-MS. (**a**,**o**) TEM images, (**b**–**e**,**p**–**s**) top-view elemental mapping images, and side-view atomic-resolution HAADF-STEM images (**f**,**t**) and EELS elemental maps (**g**–**j**,**u**–**x**) of BOC-MS/Pt and BOC-MS/MnO_x_. Scale bar, 500 nm, 10 Å, 600 nm and 10 Å, in **a**,**f**,**o**,**t**, respectively. (**k**) AFM image and (**l**) KPFM image of BOC-MS. Scale bar, 500 nm in **k**. (**m**) Height profiles along the lines in image (**k**,**l**). Schematic illustration of Pt (**n**) and MnO_x_ (**y**) photo-depositions on BOC-MS.

To further verify the atomic-level charge flow described above and directly image the destination of electrons and holes in the photoexcited Janus bilayer junctions, we further deposited Pt and MnO_x_ on BOC-MS under visible light. As shown in Fig. 4a–e,o–s, Pt preferred to deposit on MoS_2_ surfaces (Fig. 4n), while MnO_x_ tended to locate on the opposite surfaces, namely the (Cl_2_) end-faces of Bi_12_O_17_Cl_2_ (Fig. 4y). The Pt and MnO_x_ photo-deposition sites were further directly evidenced by the atomic-resolution HAADF-STEM images (Fig. 4f,t) and EELS elemental maps (Fig. 4g–j,u–x) of the cross-sectional atomic structures of BOC-MS photo-deposited with Pt and MnO_x_. When using H_2_AuCl_4_ as the metal precursors, Au still photo-deposited on the MoS_2_ surfaces in the Janus bilayers (Supplementary Fig. 21). This control experiment strongly supported that the spatially selective photo-depositions of Pt and MnO_x_ on the bilayers were induced not by the site-selective adsorptions of the metal precursors, but by the atomic-level charge flow driven by the selective and chemical assembly of MoS_2_ monolayers on (Bi_12_O_17_) end-faces of Bi_12_O_17_Cl_2_ monolayers via OV chemistry. Furthermore, the side-view TEM image of BOC-MS photo-deposited with MnO_x_ also directly evidenced that all the MnO_x_ nanoparticles were deposited on the (Cl_2_) end-faces of Bi_12_O_17_Cl_2_ monolayers (Supplementary Fig. 22 and Supplementary Note 10), well-matching with the results of the above HAADF-STEM image and EELS elemental maps. These photo-deposition experiments demonstrated the photogenerated charge carriers' transfer pathways in BOC-MS as follows: Electrons from e-h separation sites to (Bi_12_O_17_) end-faces to MoS_2_ and holes from e-h separation sites to (Cl_2_) end-faces. Furthermore, the Kelvin probe force microscopy (KPFM) was carried out to further verify these conclusions and directly image the distribution of the photogenerated electrons on the bilayers. As shown in Fig. 4k–m, 1L-MS displayed much lower surface potential than 1L-BOC, indicating that the photogenerated electrons from the photoexcited BOC-MS were inclined to accumulate on the 1L-MS^28^,^29^. This phenomenon could also serve as a direct evidence for the steered electron transfer from 1L-BOC to 1L-MS. Besides, the Janus distribution of electrons and holes in BOC-MS also indicated the existence of a Janus photocatalysis behaviour, including the electron-induced hydrogen evolution on MoS_2_ and the hole-mediated photo-oxidation of organic scavengers on (Cl_2_) end-faces of Bi_12_O_17_Cl_2_ (Supplementary Fig. 23).

## Discussion

Subsequently, we quantified the efficiencies of charge transportations during the above two charge flow processes to probe the origin of the superior PHE performance of BOC-MS by using the following equations (Supplementary Methods).




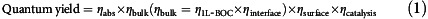







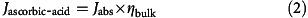







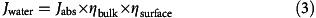




In equation (1), *η*_abs_ (set as ‘1'), *η*_bulk_, *η*_1L-BOC_, *η*_interface_, *η*_surface_ and *η*_catalysis_ are the efficiencies of photoabsorption, bulk charge separation of BOC-MS, bulk charge separation of 1L-BOC, interfacial charge flow from 1L-BOC to 1L-MS, surface charge separation and hydrogen-evolving catalysis, respectively. In equation (2) and equation (3), *J*_abs_ is the current density converted from the absorbed photons when assuming *η*_abs_=1, while *J*_ascorbic-acid_ and *J*_water_ are the photocurrent densities measured by using ascorbic acid and water as electrolytes, respectively. The establishment of equation (2) is based on the assumption that ascorbic acid oxidation could completely suppress the surface recombination and its catalytic efficiency is 1, similar with the case that Kim *et al*.^30^ used sulfite oxidation to determine the *η*_bulk_ of BiVO_4_. These experiments were performed under irradiation of 420 nm monochromatic light, which gave a *J*_abs_=0.788 mA cm^−2^. For 1L-BOC, *J*_ascorbic-acid_ was measured to be 0.725 mA cm^−2^ (Supplementary Fig. 24), so *η*_bulk_=*J*_ascorbic-acid_/*J*_abs_=0.92. This means, through the atomic-level charge separation steering by IEF, 1L-BOC enabled 92% of photogenerated electrons and holes to be transferred from the electron–hole separation sites to its (Bi_12_O_17_) and (Cl_2_) end-faces, respectively. Despite this ultrahigh *η*_bulk_, the quantum yield of 1L-BOC was still as low as 0.8%, because of its ultralow *η*_surface_ (0.8%) and poor *η*_catalysis_ (10.8%; Supplementary Fig. 24d). When 1L-MS was selectively and chemically assembled on (Bi_12_O_17_) end-faces, *η*_surface_ and *η*_catalysis_ increased by 11.4 and 4.2 times, respectively, as this oriented assembly enabled the Janus distribution of electrons and holes in BOC-MS to prevent the surface recombination and the metallic nature of 1L-MS was highly active for the hydrogen-evolving catalysis. Moreover, another crucial factor for the remarkable quantum yield (36%) of BOC-MS lied in the ultrahigh *η*_interface_ (91%) offered by the interfacial Bi–S bonds, by which the separated electrons of 1L-BOC could quickly migrate from (Bi_12_O_17_) end-faces to 1L-MS, achieving atomic-level charge flow steering from 1L-BOC to 1L-MS.

In conclusion, we have designed Janus (Cl_2_)-(Bi_12_O_17_)-(MoS_2_) bilayer junctions by assembling MoS_2_ monolayers selectively and chemically on (Bi_12_O_17_) end-faces of Bi_12_O_17_Cl_2_ monolayers via OV chemistry. This atomic-level structural and interfacial design allowed us to steer all the charge separation, transportation and consumption at the atomic level. Electrons originating from visible light-irradiated Bi_12_O_17_Cl_2_ were first driven by the IEF between (Cl_2_) and (Bi_12_O_17_) end-faces to (Bi_12_O_17_) end-faces, and further transferred via the Bi–S bonds formed between Bi_12_O_17_Cl_2_ and MoS_2_ monolayers to MoS_2_ monolayers to finally catalyse the hydrogen evolution. Meanwhile, the IEF drove holes to (Cl_2_) end-faces where the organic scavenger was oxidized. Such an atomic-level steering of charge flow offered a visible-light PHE rate of 33 mmol h^−1^ g^−1^ with a quantum efficiency of 36% at 420 nm, superior to any reported MoS_2_, or monolayer, or bismuth oxyhalide-based PHE systems. This work sheds atomic-level mechanistic insights into the flow of photogenerated electrons and holes, thus paving new ways into the exploration and design of high-performance, cost-effective photocatalysts for hydrogen evolution.

## Methods

### Preparation of bulk layered Bi_12_O_17_Cl_2_ nanosheets

First, 8 mmol of BiCl_3_ was dissolved in 80 ml of ethanol with vigorous stirring at ambient temperature. Next, the pH value of the obtained homogeneous solution was adjusted to 13.4 by dropwise adding NaOH (1 mol l^−1^) solution, yielding yellow precipitations. Then these precipitations were collected, washed thoroughly with deionized water and ethanol for several times, and dried in an oven at 50 °C under vacuum. Calcination of the collected yellow powders in muffle furnace at 450 °C for 2 h produced layered Bi_12_O_17_Cl_2_ nanosheets, which we term BOC.

### Preparation of bulk layered MoS_2_ nanosheets

Typically, 1.5 mmol of (NH_4_)_6_Mo_7_O_24_·4H_2_O and 36 mmol of thiourea were added in 75 ml of distilled water at room temperature with continuous stirring to give a transparent solution. The resulting mixture solution was then poured into a 100 ml Teflon-lined stainless autoclave. The autoclave was allowed to be heated at 200 °C for 24 h under autogenous pressure, and then air cooled to room temperature. The resulting precipitates were collected, then washed with ethanol and deionized water thoroughly, and finally dried at 50 °C under vacuum. We term the obtained sample MS.

### Preparation of Bi_12_O_17_Cl_2_ and MoS_2_ monolayers

Organolithium chemistry was used to exfoliate layered MoS_2_ (or Bi_12_O_17_Cl_2_) nanosheets into their single-layered counterpart. Briefly, intercalating lithium into the spaces between each neighbouring MoS_2_ (or Bi_12_O_17_Cl_2_) monolayer-unit was first conducted by immersing 0.2 g of layered MoS_2_ (or 0.1 g of Bi_12_O_17_Cl_2_) nanosheets in 8 ml (10 ml for Bi_12_O_17_Cl_2_) of *n*-butyllithium (Caution: *n*-butyllithium is highly pyrophoric.) under argon atmosphere for 72 h at 100 °C. The suspension was then washed with hexane for several times to remove the excess of *n*-butyllithium. The collected nanosheets with lithium intercalation were re-dispersed in distilled water at 1 mg ml^−1^ and sonicated at a low-power sonic bath (40 W) for 40 min to obtain exfoliated nanosheets. After centrifuging the resultant dispersions at 8,000 r.p.m. (8,000 r.p.m. for MoS_2_ and 6,000 r.p.m. for Bi_12_O_17_Cl_2_) for 10 min, the supernatants were collected, removing the unsuccessfully exfoliated nanosheets. The collected supernatants were centrifuged at 12,000 r.p.m for 10 min for several times to remove excess impurity, producing single-layered MoS_2_ and Bi_12_O_17_Cl_2_ nanosheets with yields of ∼47 and 26%, respectively. We term the as-prepared MoS_2_ and Bi_12_O_17_Cl_2_ monolayers 1L-MS and 1L-BOC, respectively.

It should be noteworthy that the surface of 1L-BOC was rich in oxygen vacancies, because, during the lithium-intercalation process, the organic ligands in *n*-butyllithium would interact strongly with lattice oxygen atoms on Bi_12_O_17_Cl_2_ to form coordinate bonds, and during the ultrasonication-mediated exfoliation process, the organic ligands escaped from the interior of Bi_12_O_17_Cl_2_, which inevitably removed a fraction of oxygen atoms from the lattice, leading to the occurrence of surface oxygen vacancies in single-layered Bi_12_O_17_Cl_2_ nanosheets. Calcination of 1L-BOC with oxygen-vacancy concentrations of 11% in air at 200 °C for 30 min and at 300 °C for 6 h produced single-layered Bi_12_O_17_Cl_2_ nanosheets with oxygen-vacancy concentrations of 5.3% and 0, which we call 1L-BOC-1 and 1L-BOC-2.

### Assembling monolayers of Bi_12_O_17_Cl_2_ and MoS_2_

An OV-directed assembly strategy was used to assemble monolayers of MoS_2_ and Bi_12_O_17_Cl_2_. First, 10 mg of MoS_2_ monolayers were dispersed in 30 ml of distilled water under ultrasonication to obtain transparent solution **A**. 40 mg of oxygen-deficient Bi_12_O_17_Cl_2_ monolayers were dispersed in a three-neck flask containing 120 ml of distilled water under ultrasonication to obtain transparent solution **B**. Then the solution **A** was dropwise added to the solution **B** under vigorous stirring at ambient temperature to give homogeneous mixture solution. Next, the above mixture was deoxygenated by bubbling argon gas at room temperature for 60 min, subsequently refluxed at 80 °C for 2 h under a stirring rate of 200 r.p.m., and finally air cooled to room temperature. The resulting suspension were first centrifuged at 3,000 r.p.m. for 5 min to remove the aggregated precipitations, and further centrifuged at 12,000 r.p.m. for 10 min to obtain the assemble of MoS_2_ and Bi_12_O_17_Cl_2_ monolayers, which we call BOC-MS.

We treated the synthesized 1L-BOC, 1L-MS and BOC-MS by many times of water–ethanol washing and plasma treatment to eliminate the surface-adsorbed impurities. Furthermore, when measuring their heights via AFM, we diluted the concentrations of the measured solutions to a considerably low level so as to maintain the nanosheets' surface as flat as possible, which would avoid the wrinkling or twisting.

### Characterization

The powder X-ray diffraction patterns of the samples were recorded on a Bruker D8 Advance diffractometer with monochromatized Cu Kα radiation (*λ*=0.15418, nm). The powders were deposited on copper grids with carbon support films for electron microscopy observation. TEM and HRTEM (high-resolution TEM) observations were performed on Hitachi H-7650 and JEOL-2010F with an acceleration voltage of 200 kV. The atomic-resolution HRTEM images and EELS elemental mapping images were investigated by aberration-corrected STEM and EELS carried out in a Nion UltraSTEM (Nion) microscope operating at 200 keV and equipped with a cold-field emission gun, a third-generation C3/C5 aberration corrector and a Gatan Enfinium EEL spectrometer. Energy dispersive X-ray spectroscopy were carried out using a JEM-ARM 200F Atomic-Resolution Analytical Microscope operating at an accelerating voltage of 200 kV. Elemental mappings were collected by a Gatan GIF Quantum 965 instrument. Ultraviolet–visible diffused reflectance spectra of the samples were obtained for the dry-pressed film samples with using a ultraviolet–visible spectrophotometer (UV-2550, Shimadzu, Japan) with BaSO_4_ as the reflectance standard. Chemical compositions and states were analysed using XPS (Thermo Scientific ESCLAB 250Xi). All binding energies were referenced to the C 1 s peak (284.6 eV) arising from the adventitious carbon. Atomic concentrations were calculated by normalizing peak areas to the elemental sensitivity factor. Raman measurements were carried out by a confocal laser micro-Raman spectrometer (Thermo DXR Microscope, USA). The laser was 633 nm with a 5 mW. AFM and Kelvin probe force microscope measurements were carried out on an AFM instrument (SPM-9600, Shimadzu). The electrical resistivity study was corrected on a Keithley 4200 station with the computer-controlled four-probe technique. Photoluminescence emission and photoluminescence decay spectra were recorded at room temperature with a fluorescence spectrophotometer (Edinburgh Instruments, FLSP-920). The femtosecond-resolved TA spectra were performed using a modified ExciPro pump–probe spectrometer (CDP) in connection with an amplified femtosecond laser system (Coherent). Electron paramagnetic resonance spectra were performed on a Bruker EMX EPR Spectrometer (Billerica, MA). The Bi:O:Cl molar ratios in Bi_12_O_17_Cl_2_ monolayers were detected by IRIS (INTREPID 2) inductively coupled plasma atomic emission spectrometry. The 13C NMR spectra were recorded on a Bruker AVANCE III 600M system. The Bi L-edge X-ray absorption fine structure spectroscopy was carried out at BL 14W1 beamline at the Shanghai Synchrotron Radiation Facility (SSRF) China. Surface photovoltage spectroscopy was recorded on a lock-in amplifier (SR830-DSP, Stanford Research Systems) synchronized with a light chopper (SR540).

### Photocatalytic hydrogen evolution

The photocatalytic hydrogen production experiments were performed at ambient temperature and atmospheric pressure using 300 W Xe arc lamp as the light source. In all, 10 mg of photocatalyst was dispersed in 80 ml of aqueous solution containing 0.3 M ascorbic acid in a 120 ml Pyrex flask. Before irradiation, the suspensions were bubbled with nitrogen for 30 min to remove the dissolved oxygen and to ensure the anaerobic conditions of the reaction system. During the whole reaction process, the aqueous solution with photocatalyst was continuously stirred by a magnetic stirrer. The generated hydrogen gas was analysed with an online GC (C36880-14, RESTEK) equipped with a thermal conductivity detector, where Ar was used as a carrier gas.

### Theoretical calculations

Theoretical calculations were performed using density functional theory as implemented in the VASP code. 3D periodic boundary conditions were used to approximate an infinite solid. Exchange-correlation effects were described through the generalized gradient approximation, within the Perdew–Burke–Ernzerhof formalism. The core electrons (Bi:(Xe), Cl:(Ne), O:(He)) were treated within the projector augmented wave method. The energy cutoff is set to be 520 eV, and the atomic positions are allowed to relax until the energy and force are <10^−4^ eV and 10^−3^ eV Å^−1^, respectively.

## Additional information

**How to cite this article:** Li, J. *et al*. Superior visible light hydrogen evolution of Janus bilayer junctions via atomic-level charge flow steering. *Nat. Commun.* 7:11480 doi: 10.1038/ncomms11480 (2016).

## Supplementary Material

Supplementary Information Supplementary Figures 1–25, Supplementary Tables 1–4, Supplementary Notes 1–11, Supplementary Methods and Supplementary References.
