# Supplementary material for: Superior visible light hydrogen evolution of Janus bilayer junctions via atomic-level charge flow steering
Source: Nat Commun. 2016 May 9;7:11480. doi: 10.1038/ncomms11480 (PMC4865814; doi:10.1038/ncomms11480)
Supplement: Supplementary Information — Supplementary Figures 1–25, Supplementary Tables 1–4, Supplementary Notes 1–11, Supplementary Methods and Supplementary References. [file ncomms11480-s1.pdf]

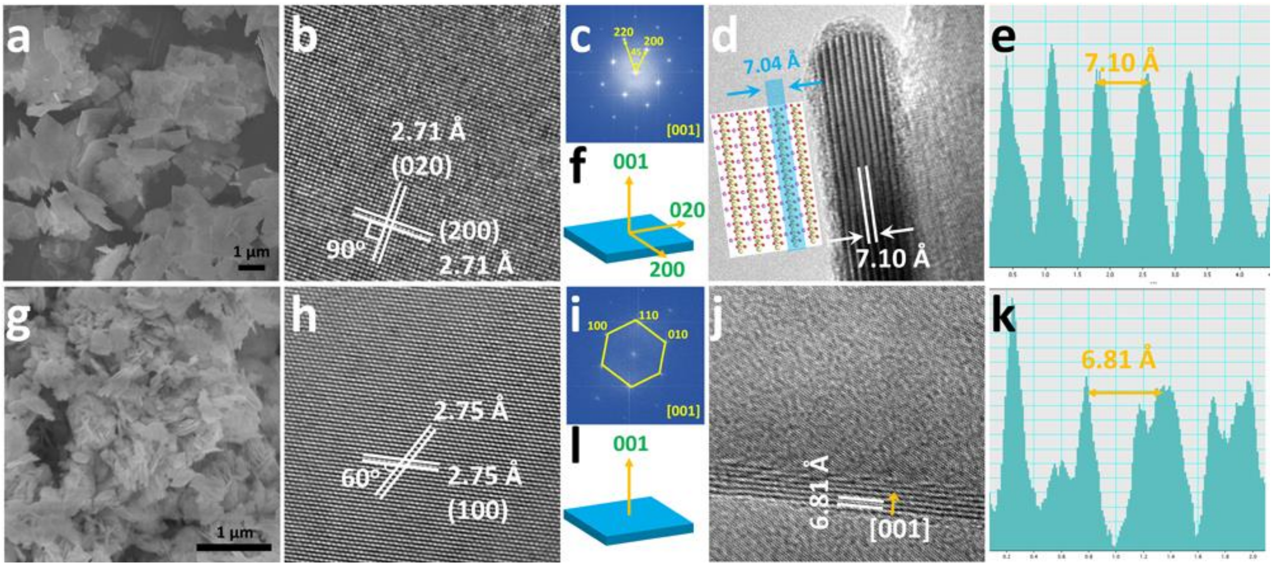

Supplementary Figure 1. Characterizations of bulk layered $Bi_{12}O_{17}Cl_2$ nanosheets (BOC) and bulk layered MoS_2 nanosheets (MS). (**a**, **g**) SEM images, (**b**, **h**) top-view HRTEM images, (**c**, **i**) SAED patterns, (**d**, **j**) side-view HRTEM images, and (**f**, **l**) the crystal orientations of BOC (**a-f**) and MS (**g-l**). The inset in **d** is the crystal structure of bulk layered $Bi_{12}O_{17}Cl_2$. (**e**, **k**) Intensity profiles along the yellow arrows in **d** and **j**, respectively.

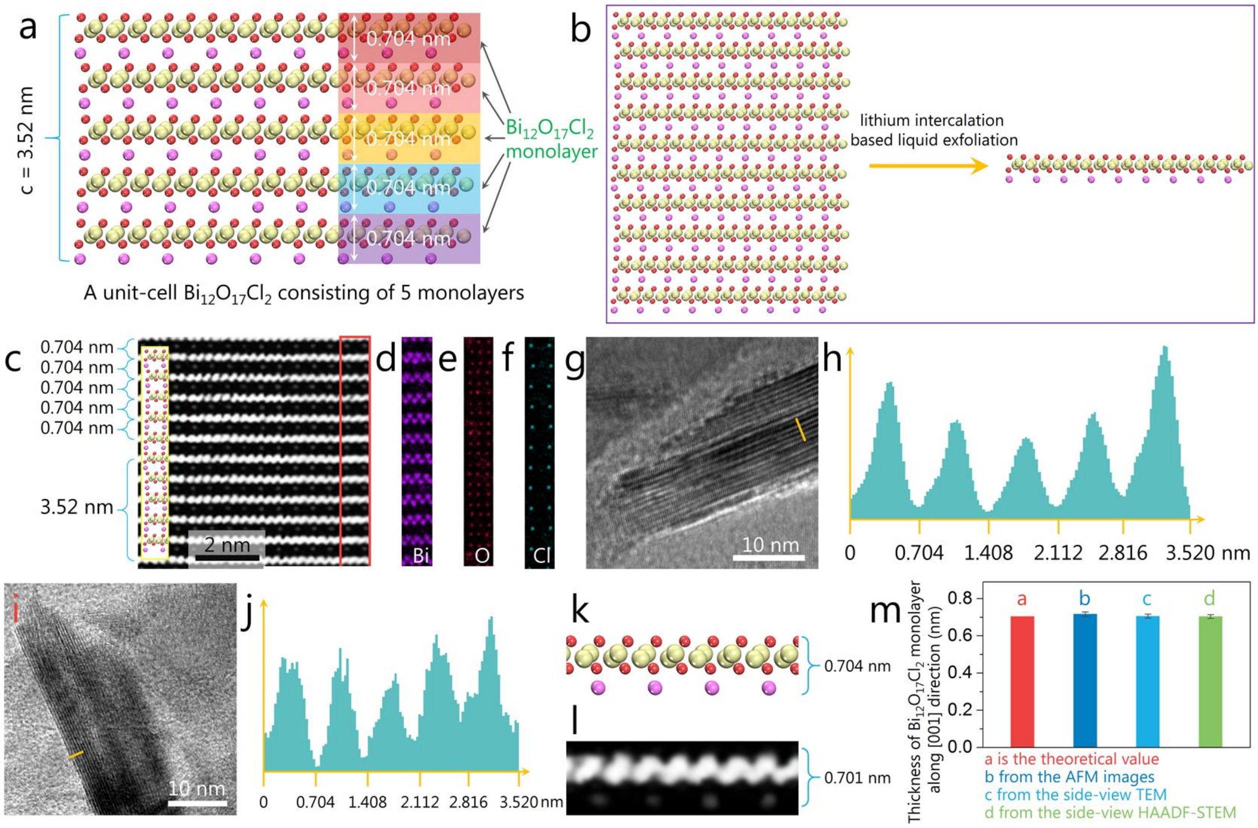

Supplementary Figure 2. The thickness of $Bi_{12}O_{17}Cl_2$ monolayer. (**a**) A unit cell $Bi_{12}O_{17}Cl_2$ (c = 3.52 nm) consisting of five $Bi_{12}O_{17}Cl_2$ monolayers. In each unit cell, the arrangements of the constituent five $Bi_{12}O_{17}Cl_2$ monolayers are along c axis; The Cl atoms in each $Bi_{12}O_{17}Cl_2$ monolayers were not in the same line along c axis. (**b**) Schematic illustration of the lithium intercalation based liquid exfoliation of bulk layered $Bi_{12}O_{17}Cl_2$ into $Bi_{12}O_{17}Cl_2$ monolayers. (**c**) The side-view atomic-resolution HAADF-STEM image and (**d-f**) the corresponding EELS elemental maps of bulk layered $Bi_{12}O_{17}Cl_2$ nanosheets. (**g**, **i**) The side-view HRTEM images of bulk layered $Bi_{12}O_{17}Cl_2$ nanosheets. (**h**, **j**) Intensity profiles along the yellow lines in **g** and **i**, respectively. (**k**) The lateral crystal structure of $Bi_{12}O_{17}Cl_2$ monolayer. (**l**) The side-view atomic-resolution HAADF-STEM image of 1L-BOC. (**m**) Comparison of the thicknesses of $Bi_{12}O_{17}Cl_2$ monolayer calculated from the theoretical model, AFM images, side-view TEM images, and side-view atomic-resolution HAADF-STEM image. The error bars in **m** represent the standard deviations.

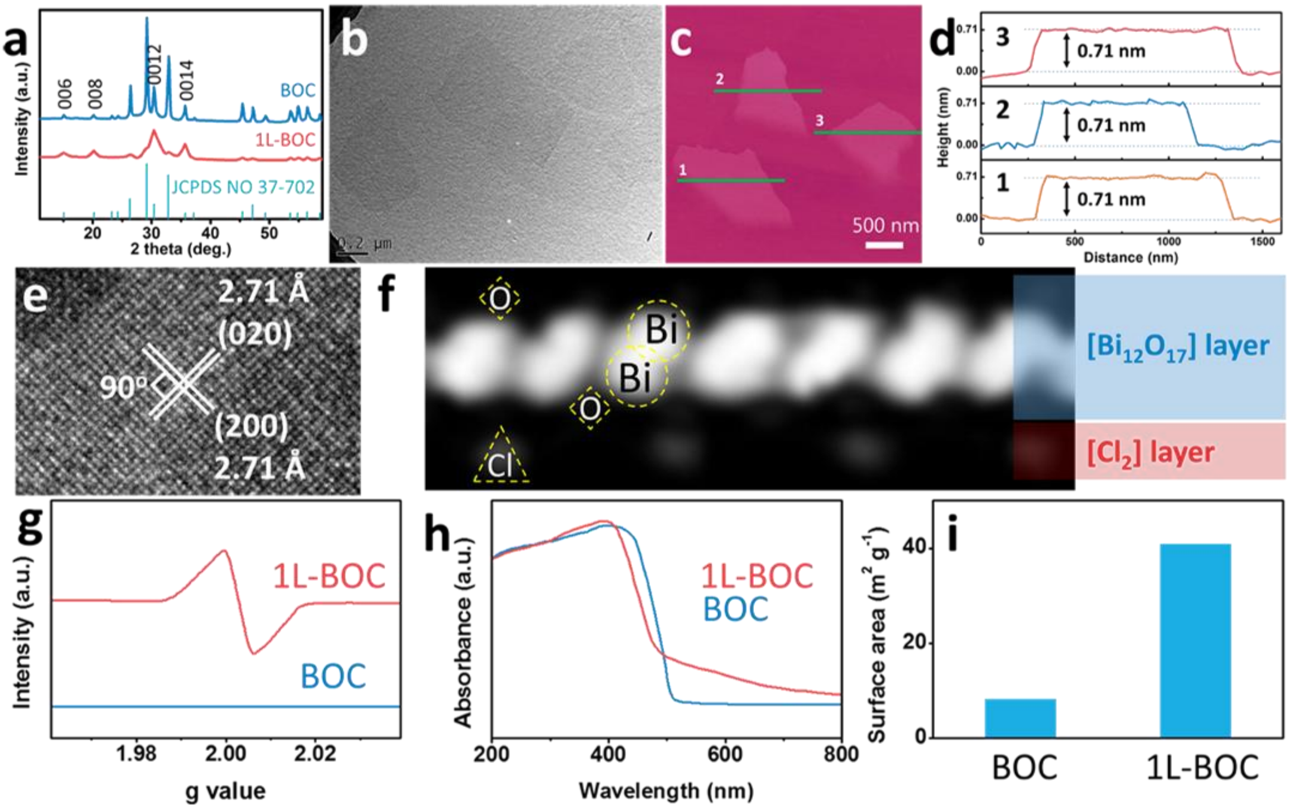

Supplementary Figure 3. Characterizations of $Bi_{12}O_{17}Cl_2$ monolayers (1L-BOC). (**a**) XRD pattern of BOC and 1L-BOC. (**b**) TEM image, (**c**) AFM image, (**d**) the height profiles (along the lines in **c**), (**e**) top-view HRTEM image, and (**f**) side-view atomic-resolution HAADF-STEM image of 1L-BOC. (**g**) EPR spectra, (**h**) DRS spectra, and (**i**) surface areas of BOC and 1L-BOC.

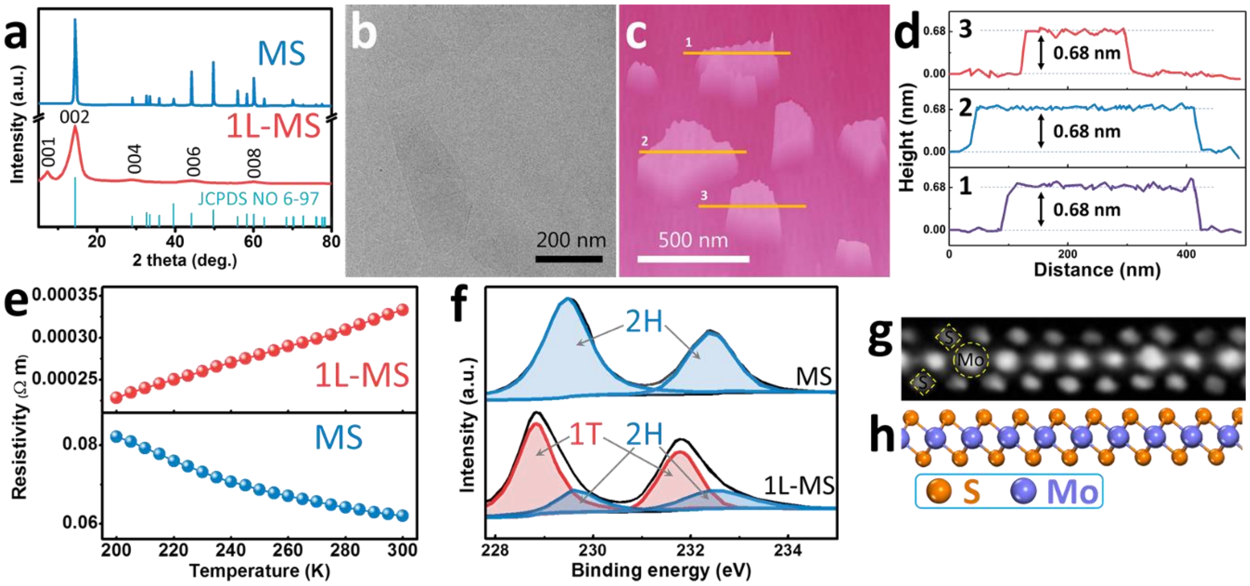

Supplementary Figure 4. Characterizations of MoS_2 monolayers (1L-MS). (**a**) XRD pattern of MS and 1L-MS. (**b**) TEM image, (**c**) AFM image, and (**d**) the height profiles (along the lines in **c**) of 1L-MS. (**e**) Temperature-dependent resistivities and (**f**) high resolution Mo 3d XPS spectra of MS and 1L-MS. (**g**) Side-view atomic-resolution HAADF-STEM image of 1L-MS. (**h**) The lateral crystal structure of 1T metallic MoS_2 monolayer.

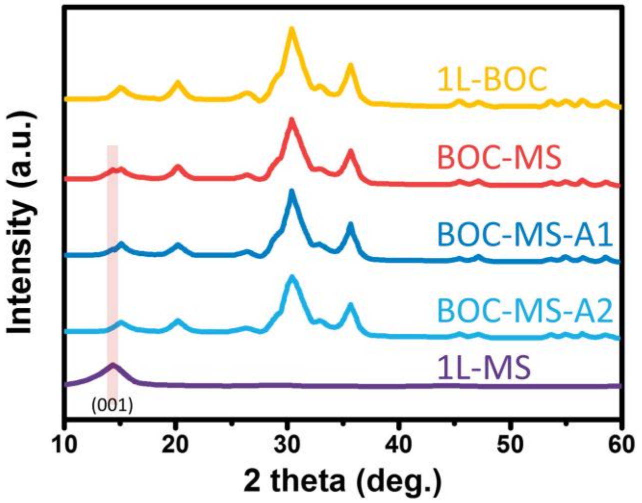

Supplementary Figure 5. XRD pattern of the Janus bilayer junctions synthesized by assembling metallic MoS_2 monolayers with $Bi_{12}O_{17}Cl_2$ monolayers of different concentrations of surface oxygen vacancies. Calcination of 1L-BOC with oxygen vacancy concentration of 11% in air at 200 ^{o}C for 30

minutes and at 300 °C for 6 h produced 1L-BOC-1 and 1L-BOC-2 with oxygen vacancy concentrations of 5.3% and 0, respectively. OV-directed assembly strategy was employed to assemble 1L-BOC-1 (or 1L-BOC-2) with 1L-MS to synthesize BOC-MS-A1 (or BOC-MS-A2).

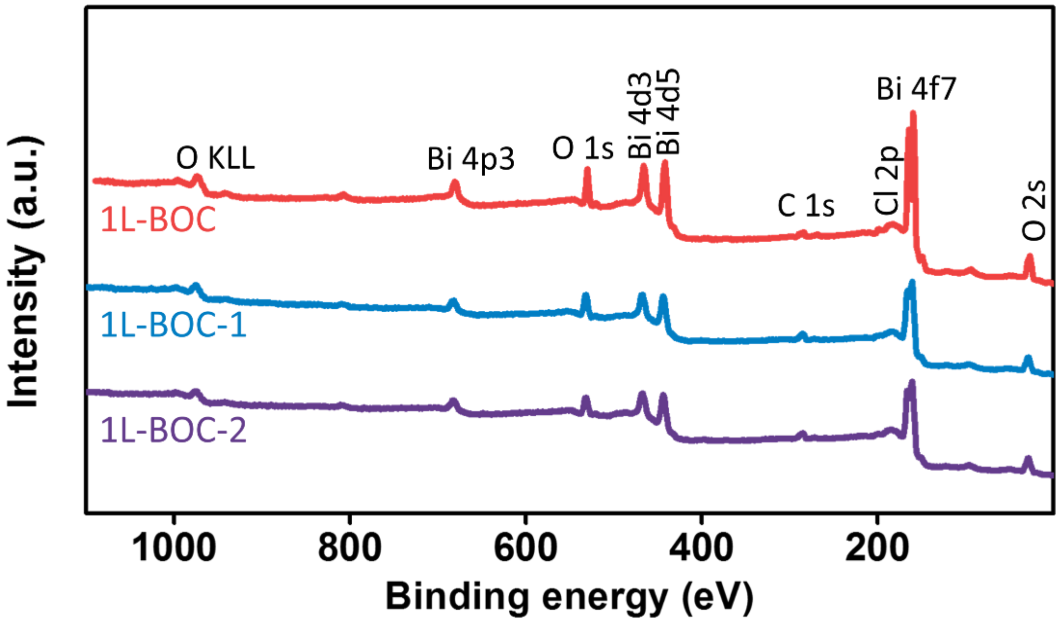

Supplementary Figure 6. Characterizations of $Bi_{12}O_{17}Cl_2$ monolayers with different OV concentrations. XPS spectra (0 s Ar^+ sputtering) of $Bi_{12}O_{17}Cl_2$ monolayers with different concentrations of surface oxygen vacancies. 1L-BOC-1 and 1L-BOC-2 were synthesized by calcination of 1L-BOC in air at 200 °C for 30 minutes and at 300 °C for 6 h, respectively.

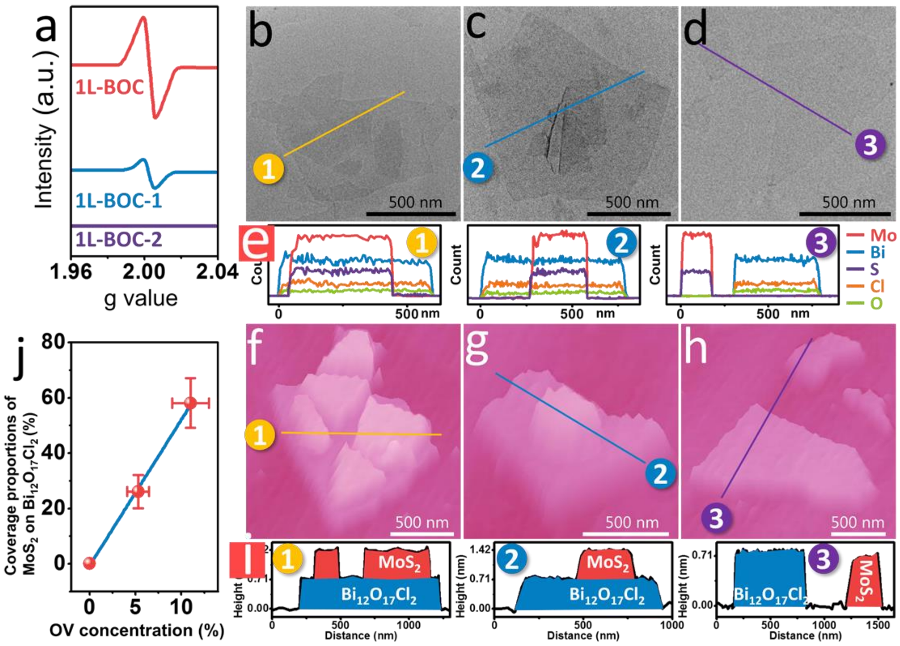

Supplementary Figure 7. Exploration of the role of oxygen vacancies confined on single-layered $Bi_{12}O_{17}Cl_2$ surfaces in assembling $Bi_{12}O_{17}Cl_2$ and MoS_2 monolayers. 1L-BOC-1 and 1L-BOC-2 were synthesized by calcination of $Bi_{12}O_{17}Cl_2$ monolayers (1L-BOC) in air at 200 ^{o}C for 30 minutes and at 300 ^{o}C for 6 h, respectively. BOC-MS-A1 (or BOC-MS-A2) were synthesized by assembling MoS_2 monolayers (1L-MS) with 1L-BOC-1 (or 1L-BOC-2). (**a**) EPR spectra of 1L-BOC, 1L-BOC-1, and 1L-BOC-2, demonstrating the calcination-driven variation of oxygen vacancy concentration in $Bi_{12}O_{17}Cl_2$ monolayers. (**b-d**) TEM images, (**e**) EDS line-scanning profiles, (**f-h**) AFM images, and (**i**) height profiles of BOC-MS (**b**, **e1**, **f**, **i1**), BOC-MS-A1 (**c**, **e2**, **g**, **i2**) and BOC-MS-A2 (**d**, **e3**, **h**, **i3**). (**j**) Correlation of the oxygen vacancy concentration with the surface coverage proportions of MoS_2 on $Bi_{12}O_{17}Cl_2$, identifying oxygen vacancies as the driving force to assemble $Bi_{12}O_{17}Cl_2$ and MoS_2 monolayers. The oxygen vacancy concentration (C_{ov}) was calculated by the equation: $C_{ov} = x/(12 + 17 + 2) = x/31$, where x represents the proportion of the oxygen atoms absent within the framework of $Bi_{12}O_{17}Cl_2$ monolayer (the corresponding chemical formula was $Bi_{12}O_{17-x}Cl_2$). The error bars in **j** represent the standard deviations.

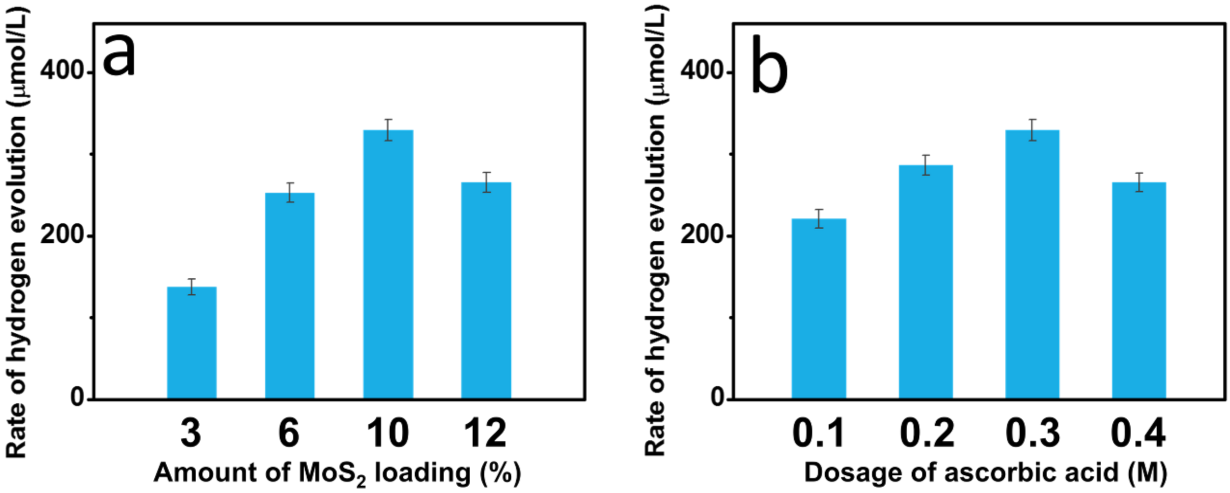

Supplementary Figure 8. Optimal reaction conditions for PHE. (**a**) Rate comparison of photocatalytic hydrogen evolution over the Janus bilayer junctions containing different amounts of MoS_2 (Reaction conditions: 10 mg assemblies; visible light, λ > 420 nm; 0.3 mol L^{-1} ascorbic acid). (**b**) Rate comparison of photocatalytic hydrogen evolution over the Janus bilayer junctions using different concentrations of ascorbic acid (Reaction conditions: 10 mg catalysts; visible light, λ > 420 nm; 10 wt % MoS_2). The error bars in **a** and **b** represent the standard deviations of five independent PHE measurements.

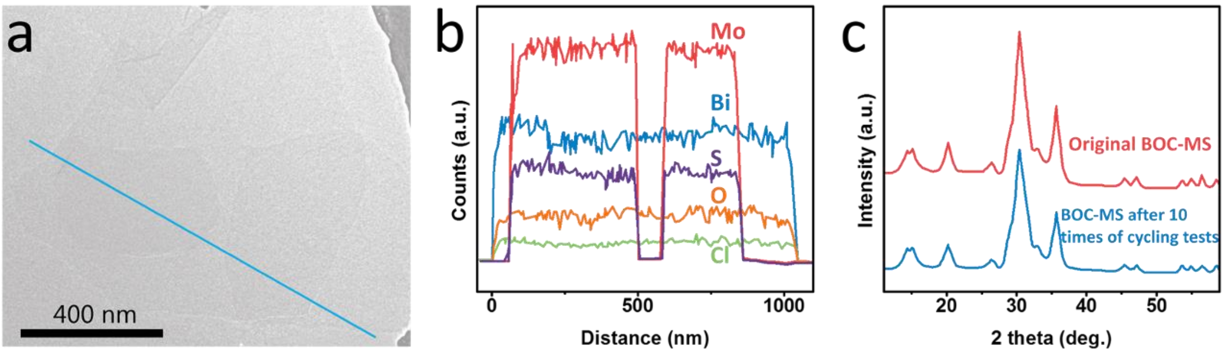

Supplementary Figure 9. Photo-stability and structural robustness of the Janus bilayer junctions. (**a**) TEM image of BOC-MS after 100 h (10 times of successive cycles) of photocatalytic

experiments. (**b**) EDS line-scanning profile along the blue line in **a**. (**c**) XRD pattern of original BOC-MS and BOC-MS after 100 h of photocatalytic experiments.

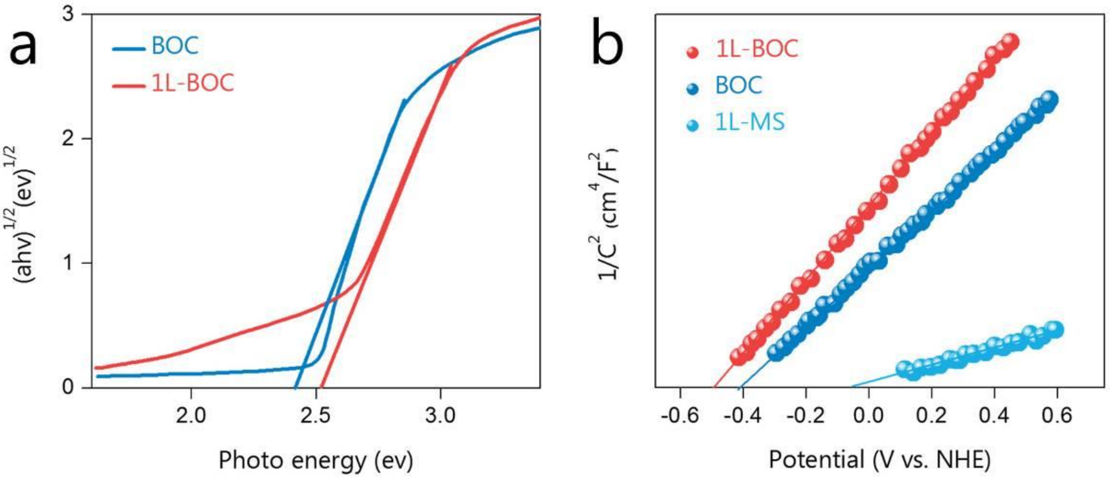

Supplementary Figure 10. Band alignments of BOC, 1L-BOC, and 1L-MS. (**a**) Plot of the transformed Kubelka–Munk function vs the light energy for BOC and 1L-BOC. (**b**) Mott-Schottky plots of BOC, 1L-BOC, and 1L-MS.

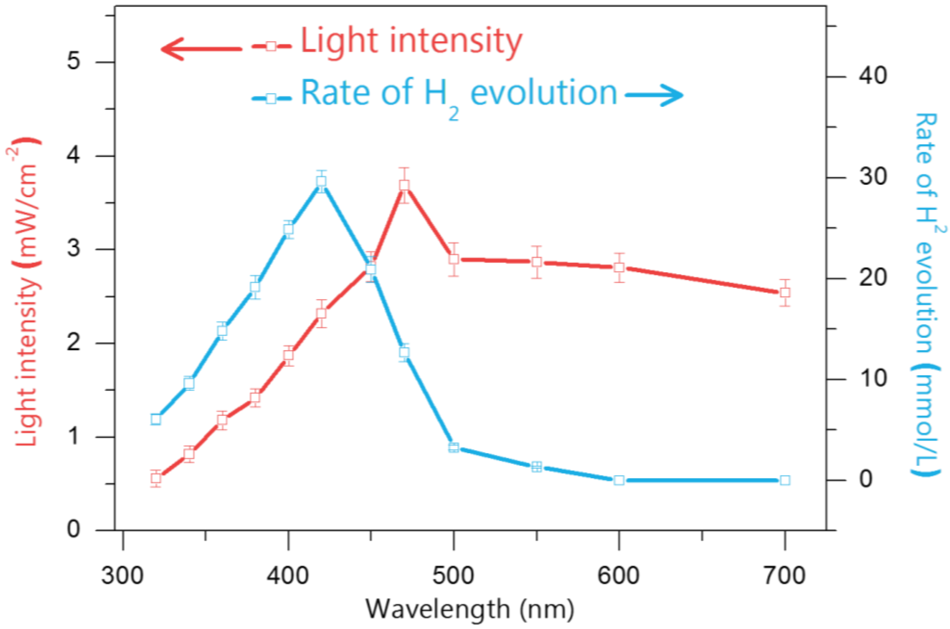

Supplementary Figure 11. Parameters for quantum yield calculation. Measurements of the intensities of the incident monochromatic light with given wavelengths (320, 340, 360, 380, 400, 420, 450, 470, 500, 550, 600, and 700 nm) and of photocatalytic hydrogen evolution over the Janus

bilayer under the irradiation of monochromatic light with given wavelengths (320, 340, 360, 380, 400, 420, 450, 470, 500, 550, 600, and 700 nm). The error bars represent the standard deviations of five independent measurements.

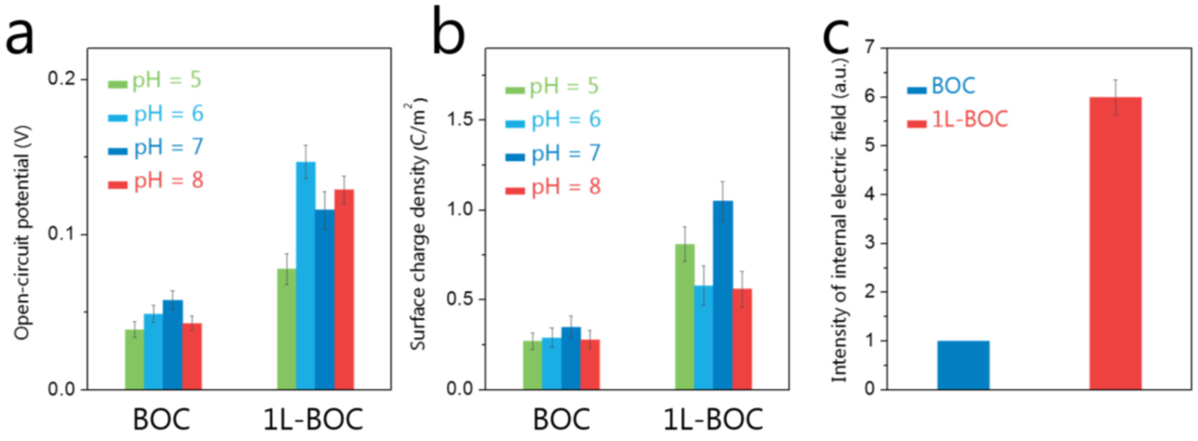

Supplementary Figure 12. Parameters for IFF magnitude calculation. (**a**) Open-circuit potentials and (**b**) surface charge densities of BOC and 1L-BOC. (**c**) Comparison of the IEF magnitude of BOC and 1L-BOC. All error bars represent the standard deviations of five independent measurements.

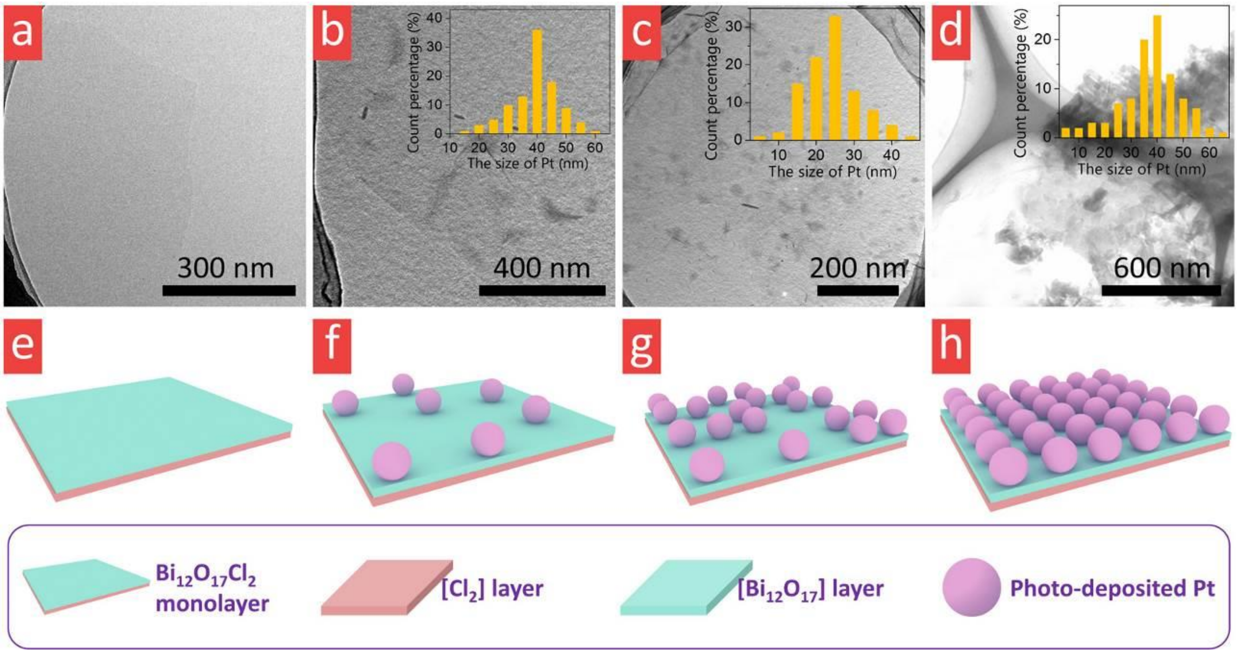

Supplementary Figure 13. Characterizations of $Bi_{12}O_{17}Cl_2$ monolayers photo-deposited with Pt. (**a-d**) TEM images and (**e-h**) schematic illustration of the morphologies of 1L-BOC photodeposited with 0 (**a**, **e**), 9 (**b**, **f**), 38 (**c**, **g**), and 66 (**d**, **h**) wt % Pt. Pt photo-depositions were performed using H_2PtCl_6 as precursors under visible light ($\lambda > 420$ nm).

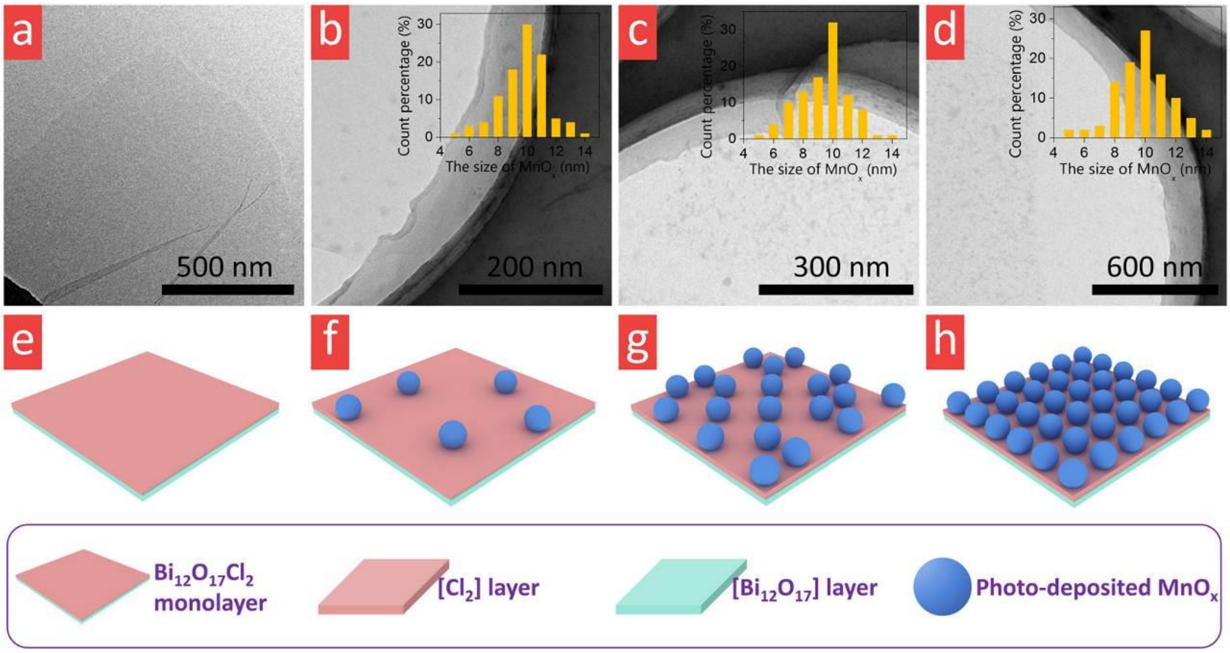

Supplementary Figure 14. Characterizations of $Bi_{12}O_{17}Cl_2$ monolayers photo-deposited with MnO_x. (**a-d**) TEM images and (**e-h**) schematic illustration of the morphologies of 1L-BOC photodeposited with 0 (**a**, **e**), 6 (**b**, **f**), 32 (**c**, **g**), and 51 (**d**, **h**) wt % MnO_x. MnO_x photo-depositions were performed using $Mn(NO_3)_2$ as precursors under visible light ($\lambda > 420$ nm).

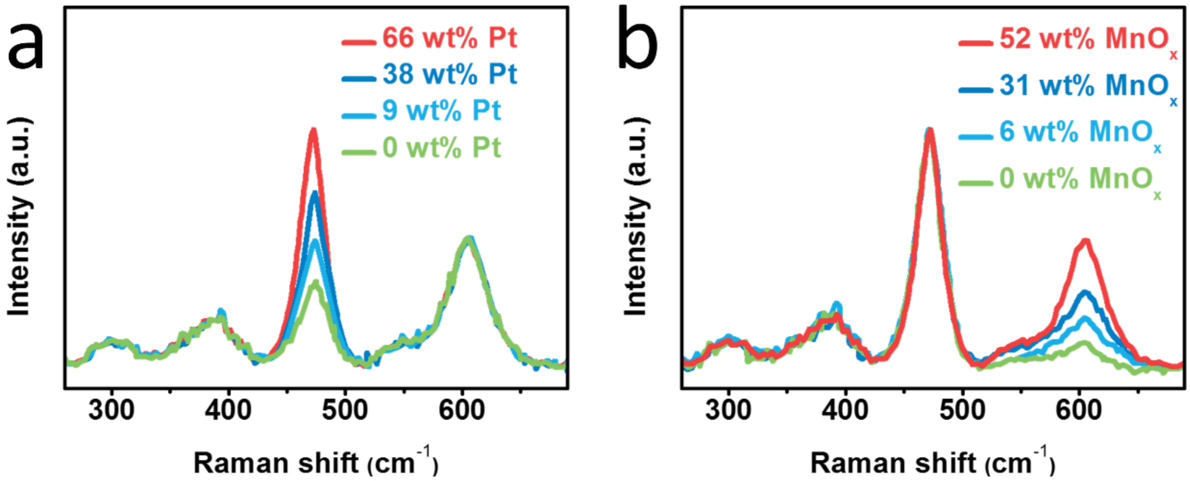

Supplementary Figure 15. Evidences for the photo-deposition sites of Pt and MnO_x on $Bi_{12}O_{17}Cl_2$ monolayers. Raman spectra of 1L-BOC photo-deposited with different amounts of Pt (**a**) and MnO_x (**b**). The characteristic bands at 473 and 605 cm^{-1} were assigned to the Bi-O stretching mode and Cl-Cl stretching mode, respectively[1-4].

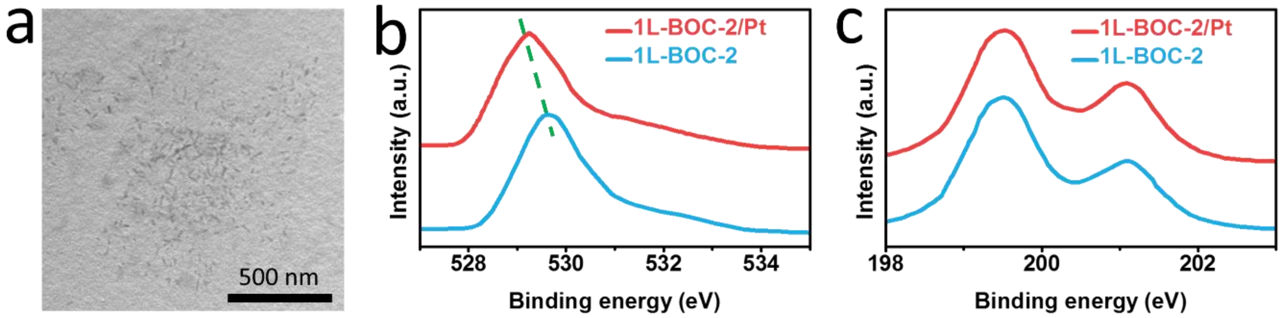

Supplementary Figure 16. Clarification that the oriented photo-depositions of Pt and MnO_x were not induced by OVs. (**a**) TEM image of 1L-BOC-2 photo-deposited with Pt (1L-BOC-2/Pt). High resolution O 1s (**b**) and Cl 2p (**c**) XPS spectra of 1L-BOC-2 and 1L-BOC-2/Pt.

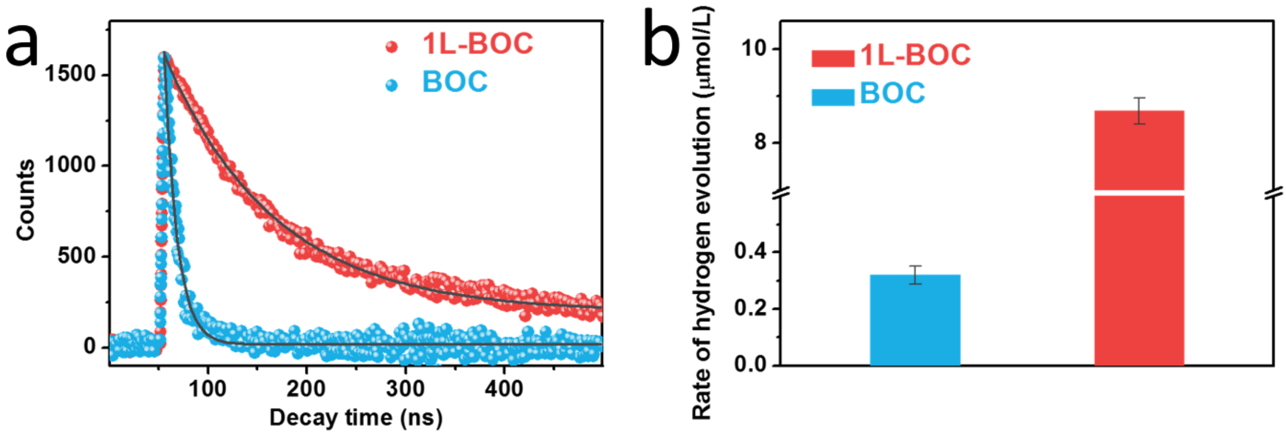

c

Sample	Decay time (ns)		Relative percentage (%)		Average lifetime (ns)	R^2
	τ_1	τ_2	I_1	I_2		
1L-BOC	11.8	169	28	72	136	0.982
BOC	5.2	12.7	47	53	9	0.991

Supplementary Figure 17. Charge dynamics and PHE activities of BOC and 1L-BOC. (**a**) Time-resolved photoluminescence decay curves of BOC and 1L-BOC recorded at 850 nm (A picosecond pulsed light emitting diode laser (400 nm) with pulse width of 120 ps was used for excitation). (**b**) Rate comparison of photocatalytic hydrogen evolution over BOC and 1L-BOC. Reaction conditions: 10 mg BOC (or 1L-BOC); visible light, $\lambda > 420$ nm; 0.3 mol L^{-1} ascorbic acid. The error bars in **b** represent the standard deviations of five independent PHE measurements. (**c**) Summary of the fitting parameters for the time-resolved photoluminescence decay curves of BOC and 1L-BOC. The decay curves were fitted based on the equation: $y = y_0 + A_1\exp(-t/\tau_1) + A_2\exp(-t/\tau_2)$. The average lifetime was calculated by using the equation: $\tau_t = (A_1\tau_1^2 + A_2\tau_2^2)/(A_1\tau_1 + A_2\tau_2)$.

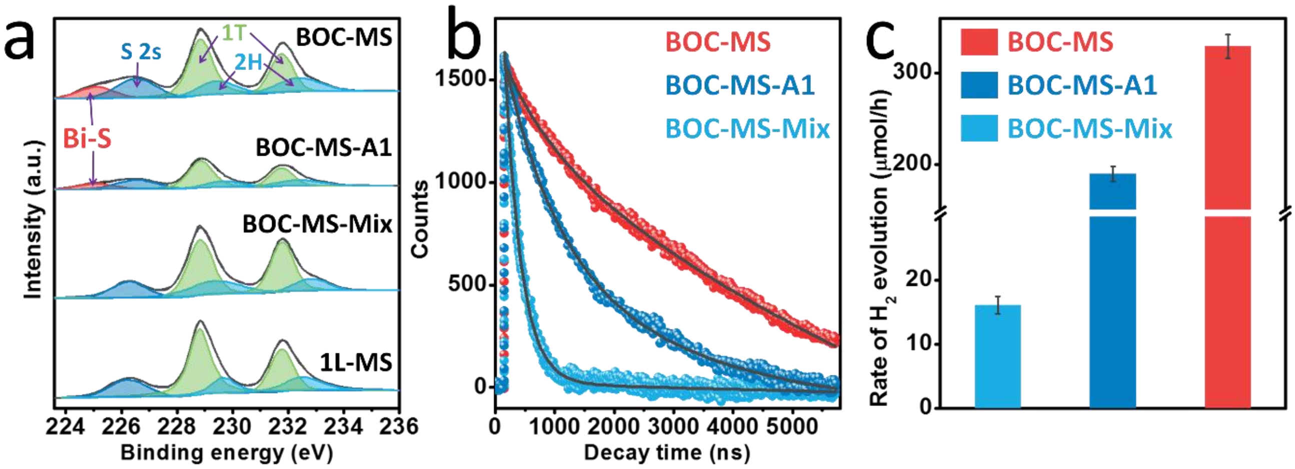

d

Sample	Decay time (ns)			Relative percentage (%)			Average lifetime (ns)	R^2
	τ_1	τ_2	τ_3	I_1	I_2	I_3		
BOC-MS	123	2971	4723	5	39	56	3446	0.992
BOC-MS-A1	69	1957	2965	36	53	11	1368	0.993
BOC-MS-Mix	28	182	1001	42	52	6	165	0.996

Supplementary Figure 18. Evidences of Bi-S bonds and their effects on charge dynamics and PHE activities. (**a**) Comparison of XPS spectra of 1L-MS, BOC-MS-A1, BOC-MS and BOC-MS-Mix. (**b**) Time-resolved photoluminescence decay curves of BOC-MS-A1, BOC-MS and BOC-MS-Mix recorded at 850 nm (A picosecond pulsed light emitting diode laser (400 nm) with pulse width of 120 ps was used for excitation). (**c**) Rate comparison of photocatalytic hydrogen evolution over BOC-MS-A1, BOC-MS and BOC-MS-Mix. Reaction conditions: 10 mg BOC-MS (or BOC-MS-Mix, or BOC-MS-A1); visible light, $\lambda > 420$ nm; 0.3 mol L^{-1} ascorbic acid. The error bars in **c** represent the standard deviations of five independent PHE measurements. (**d**) Summary of the fitting parameters for the time-resolved photoluminescence decay curves of BOC and 1L-BOC. The decay curves were fitted based on the equation: $y = y_0 + A_1\exp(-t/\tau_1) + A_2\exp(-t/\tau_2) + A_3\exp(-t/\tau_3)$. The average lifetime was calculated by using the equation: $\tau_t = (A_1\tau_1^2 + A_2\tau_2^2 + A_3\tau_3^2)/(A_1\tau_1 + A_2\tau_2 + A_3\tau_3)$.

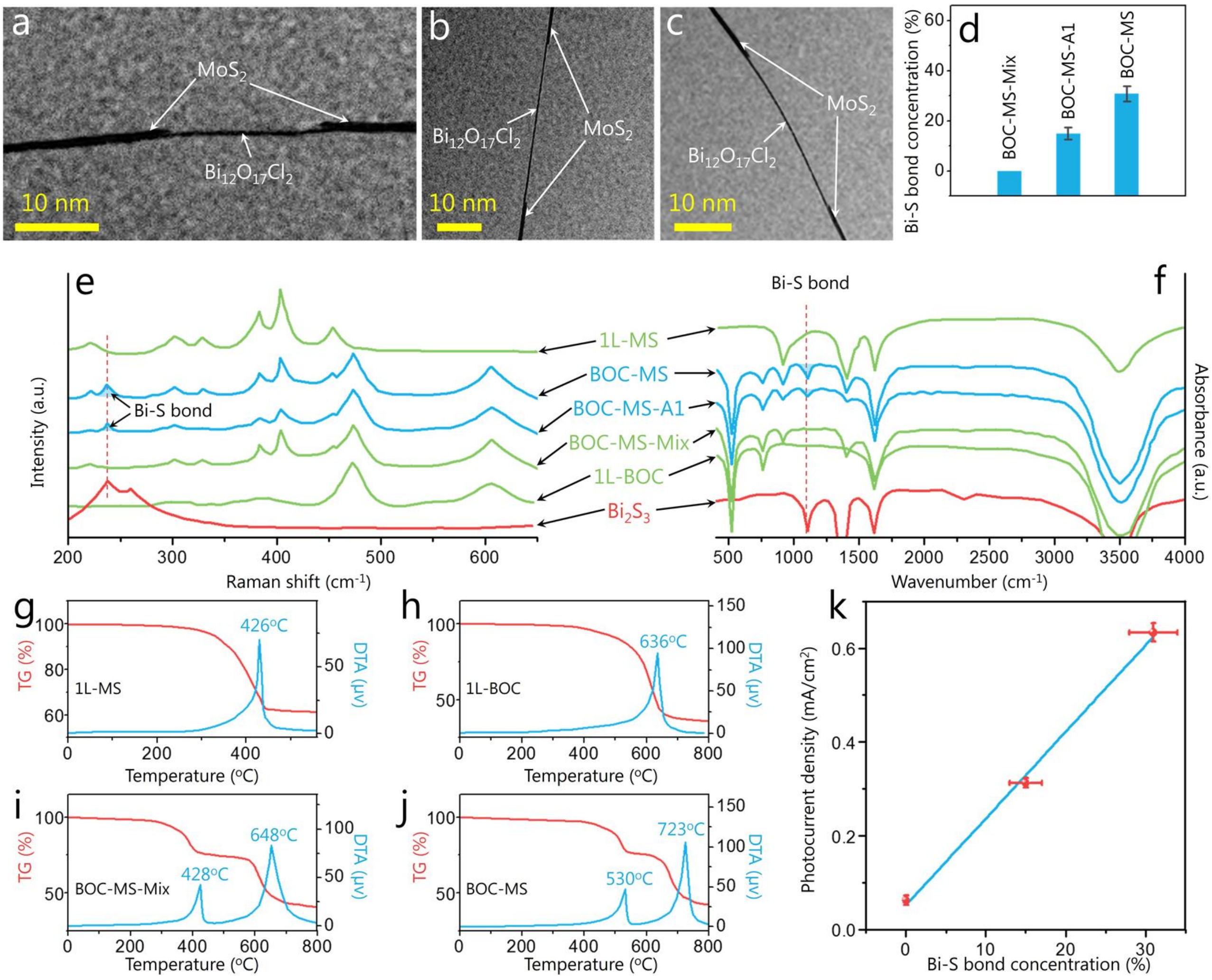

Supplementary Figure 19. Evidences for Bi-S bonds. (**a**) The magnified image of the **Figure 1m**. (**b**, **c**) The side-view TEM images of BOC-MS. (**d**) Comparison of the Bi-S bond concentrations in BOC-MS-Mix, BOC-MS-A1 (the synthesis and characterizations can be found in **Supplementary Fig. 5-7**), and BOC-MS. The Bi-S bond concentration ($C_{Bi\text{-}S}$) is calculated based on the equation of $C_{Bi\text{-}S} = N_{Bi\text{-}S}/N_{Bi}$, where $N_{Bi\text{-}S}$ is the number of Bi-S bonds and N_{Bi} is the number of Bi atoms. The error bars in **d** represent the standard deviations of five independent calculations. (**e**) Raman spectra and (**f**) FTIR spectra of 1L-MS, BOC-MS, BOC-MS-A1, BOC-MS-Mix, 1L-BOC, and Bi_2S_3. TG-DTA curves of 1L-MS (**g**), 1L-BOC (**h**), BOC-MS-Mix (**i**), and BOC-MS (**j**). (**k**) Correlation

between the Bi-S bond concentrations calculated in **Supplementary Fig. 19d** and the photocurrent densities.

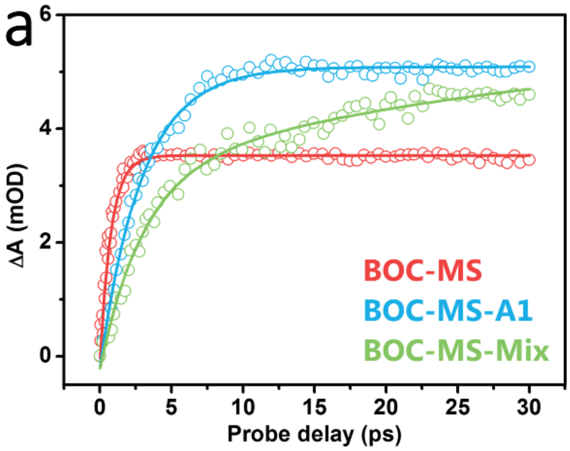

b

Sample	Decay time (ps)			Relative percentage (%)			R^2
	τ_1	τ_2	τ_3	I_1	I_2	I_3	
BOC-MS	0.68	3.59	8.25	86.59	9.32	4.09	0.998
BOC-MS-A1	1.23	4.82	10.93	49.02	22.61	28.37	0.996
BOC-MS-Mix	1.96	5.87	26.38	5.23	26.24	68.53	0.997

Supplementary Figure 20. TAS dynamics of BOC-MS, BOC-MS-A1, and BOC-MS-Mix. (**a**) Comparison of the build-up dynamics of electrons' excited-state absorption (ESA) signals (pumped at 400 nm and probed at 650 nm) of BOC-MS, BOC-MS-A1, and BOC-MS-Mix. (**b**) Summary of the fitting parameters for the ESA curves of BOC-MS, BOC-MS-A1, and BOC-MS-Mix. The curves were fitted based on the equation: $y = y_0 + A_1\exp(-t/\tau_1) + A_2\exp(-t/\tau_2) + A_3\exp(-t/\tau_3)$. The average lifetime was calculated by using the equation: $\tau_t = (A_1\tau_1^2 + A_2\tau_2^2 + A_3\tau_3^2)/(A_1\tau_1 + A_2\tau_2 + A_3\tau_3)$.

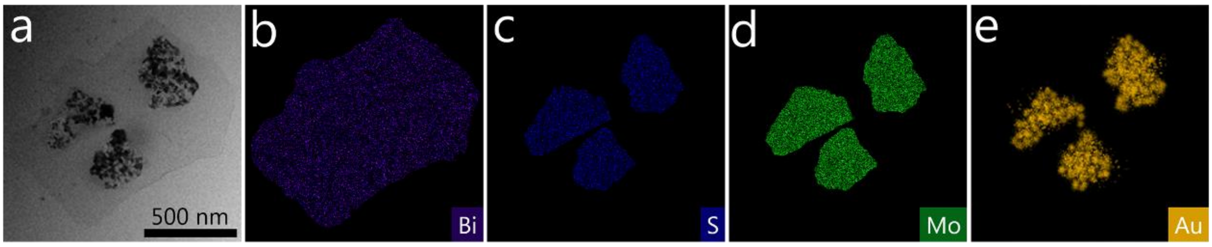

Supplementary Figure 21. (**a**) TEM images and (**b-e**) elemental mapping images of BOC-MS photo-deposited with Au.

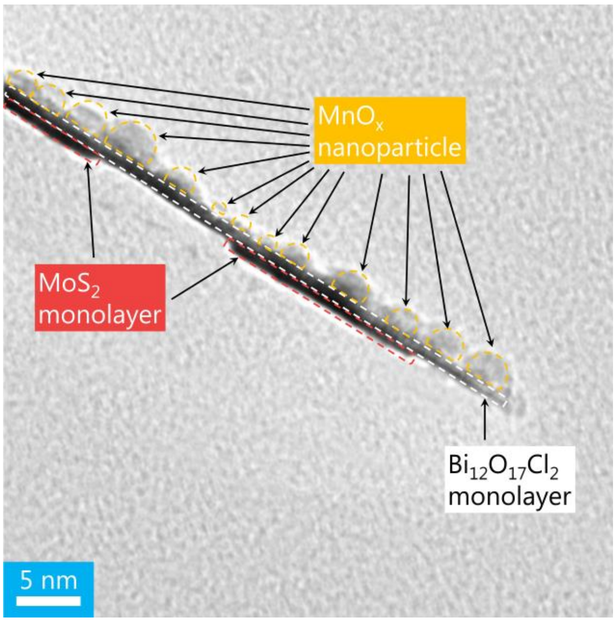

Supplementary Figure 22. The side-view HRTEM image of the Janus bilayer junctions photo-deposited with MnO_x.

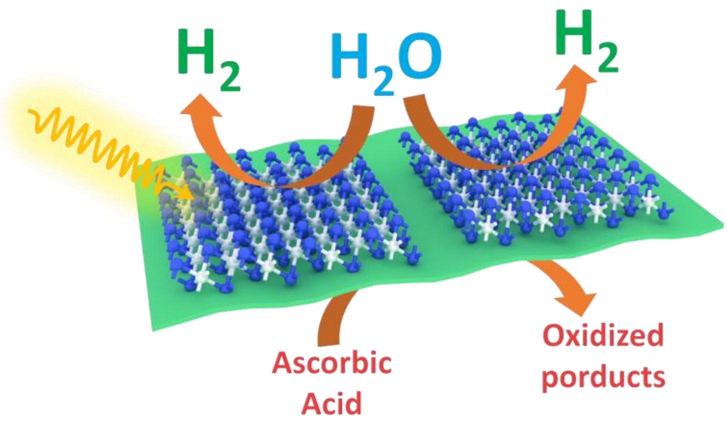

Supplementary Figure 23. Schematic illustration of the Janus-photocatalysis behavior including electron-induced hydrogen evolution on MoS_2 and hole-mediated photo-oxidation of organic scavengers on $[Cl_2]$ layers of $Bi_{12}O_{17}Cl_2$.

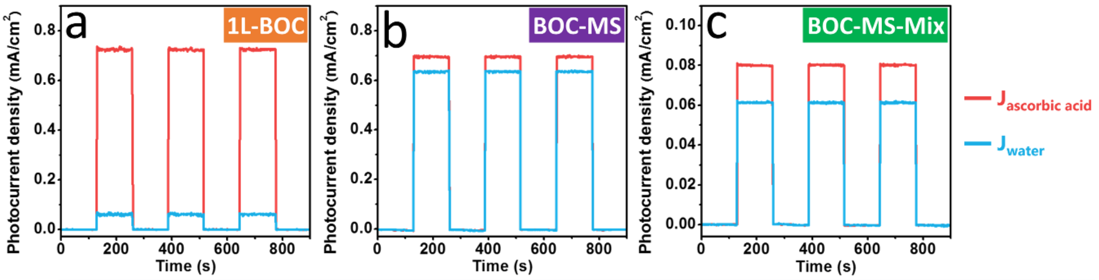

d

Sample	$J_{ascorbic\ acid}$ (mA/cm^2)	J_{water} (mA/cm^2)	η_{bulk}		$\eta_{surface}$	$\eta_{catalysis}$	Quantum yield
			$\eta_{1L\text{-}BOC}$	$\eta_{interface}$			
BOC-MS	0.696	0.633	92%	96%	91%	44.8%	36%
BOC-MS-Mix	0.08	0.061	92%	11%	76%	15.6%	1.2%
1L-BOC	0.725	0.058	92%		8%	10.8%	0.8%

Supplementary Figure 24. Transient photocurrent responses of 1L-BOC (**a**), BOC-MS (**b**), and BOC-MS-Mix (**c**). $J_{ascorbic\text{-}acid}$ and J_{water} are the photocurrent densities measured by using ascorbic acid and water as electrolytes, respectively. (**d**) Summary of the measured (or calculated) $J_{ascorbic\text{-}acid}$, J_{water}, η_{bulk} (bulk charge-separation efficiency), $\eta_{1L\text{-}BOC}$ (bulk charge-separation efficiency of 1L-BOC), $\eta_{interface}$ (efficiency of interfacial charge-flow from 1L-BOC to 1L-MS), $\eta_{surface}$ (surface charge-separation efficiency), $\eta_{catalysis}$ (hydrogen-evolving catalysis efficiency), and PHE quantum yields of 1L-BOC, BOC-MS, and BOC-MS-Mix. PHE quantum yield = $\eta_{abs} \times \eta_{bulk}$ ($\eta_{bulk} = \eta_{1L\text{-}BOC} \times \eta_{interface}$) $\times\ \eta_{surface} \times \eta_{catalysis}$; $J_{ascorbic\text{-}acid} = J_{abs} \times \eta_{bulk}$; $J_{water} = J_{abs} \times \eta_{bulk} \times \eta_{surface}$. In all the above calculations, we assumed that the absorbed photons could be completely converted into current, so the photo-absorption efficiency (η_{abs}) was set as 1. J_{abs} was obtained by the equation: $J_{abs} = (Nq)/t$, where t, N, and q were the irradiation time, the number of incident photons during the irradiation time t, and the charge of a single electron. $N = (E\lambda)/(hc)$, where E, λ, h, and c are the energy of incident photons, the wavelength of the incident monochromatic light, the Planck constant, and the light speed, respectively. The intensity of incident monochromatic light at 420 nm was measured to be ca. 2.32 mW cm^{-2}, matching well with the previously reported value[12].

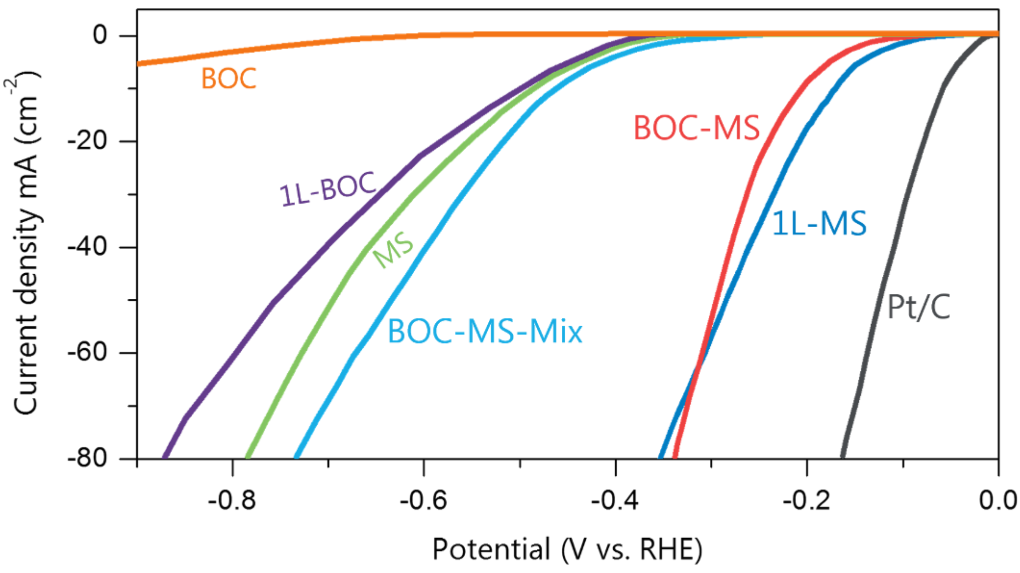

Supplementary Figure 25. Hydrogen-evolving polarization curves for Pt/C, BOC-MS, BOC-MS-Mix, 1L-BOC, MS, 1L-MS, and BOC.

Supplementary Table 1. Elemental compositions of 1L-BOC, 1L-BOC-1, and 1L-BOC-2 determined by XPS (**Supplementary Fig. 6**) and ICP, respectively.

sample	Bi:O:Cl ratio	
	Calculation from XPS	Calculation from ICP
**1L-BOC**	12:13.59:2.03	12:13.42:2.04
**1L-BOC-1**	12:15.36:2.02	12:15.47:2.05
**1L-BOC-2**	12:17.01:2.04	12:17.05:2.03

Supplementary Table 2. Comparison of photocatalytic hydrogen evolution activities of Janus $MoS_2/Bi_{12}O_{17}Cl_2$ bilayer with some representative MoS_2-based photocatalysts.

Photocatalyst	Hole scavenger	Light Source	PHE rate ($mmol \cdot h^{-1} \cdot g^{-1}$)	Reference
**Janus $MoS_2/Bi_{12}O_{17}Cl_2$ bilayer**	**Ascorbic acid**	**300 W Xenon lamp, λ > 420 nm**	**33**	**This work**
**MoS_2/CdS**	Lactic acid	300 W Xenon lamp, λ > 420 nm	5.34	J. Am. Chem. Soc. 2008, 130, 7176-7177.
**TiO_2/MoS_2/graphene**	Ethanol	350 W Xenon lamp, full spectrum	2.07	J. Am. Chem. Soc. 2012, 134, 6575-6578.
**MoS_2/C_3N_4**	Lactic acid	300 W Xenon lamp, λ > 420 nm	1.38	Angew. Chem. Int. Ed. 2013, 52, 3621-3625.
**MoS_2/graphene-oxide**	Ethanol	300 W Xenon lamp, full spectrum	$2.48 \cdot 10^{-2}$	J. Am. Chem. Soc. 2013, 135, 10286-10289.
**MoS_2/graphene/CdS**	Lactic acid	300 W Xenon lamp, λ > 420 nm	9	ACS Nano 2014, 8, 7078-7087.
**MoS_2-monolayer/CdS**	Lactic acid	300 W Xenon lamp, λ > 420 nm	1.47	J. Phys. Chem. C. 2012, 116, 25415-25424.
**$MoS_2/ZnIn_2S_4$**	Lactic acid	300 W Xenon lamp, λ > 420 nm	8.05	Appl. Catal. B 2014, 160, 614-620.
**2-D MoS_2/CdS p–n nanohybrids**	Lactic acid	300 W Xenon lamp, λ > 420 nm	6.85	Chem. Eur. J. 2014, 20, 10632-10635.
**MoS_2–graphene/CdS nanoparticles/nanorods**	Lactic acid	300 W Xenon lamp, λ > 400 nm	23.2	Chem. Commun. 2014, 50, 11004-11007.
**MoS_2/graphene/ZnS**	Na_2SO_3	300 W Xenon lamp, full spectrum	2.26	J. Mater. Chem. A 2014, 2, 3819-3827.
**MoS_2/Cu_2O**	Methanol	350 W Xenon lamp, λ > 420 nm	0.58	J. Phys. Chem. C 2014, 118, 14238-14245.

**MoS_2-monolayer/TiO_2**	Methanol	300 W Xenon lamp, λ < 400 nm	1.98	Nano Research 2015, 8, 175-183.
**MoS_2 Graphene-analogue/ZnxCd$_{1-x}$S**	Lactic acid	150 W Xenon lamp, λ > 420 nm	7.18	Catal. Sci. Technol. 2014, 4, 2650-2657.
**MoS_2-monolayer/ Eosin-Y**	Triethanolamine	100 W halogen lamp, full spectrum	30	J. Phys. Chem. C 2012, 116, 25415-25424.
**MoS_2/CdS/γ-TaON**	Na_2SO_3	300 W Xenon lamp, λ > 420 nm	3.14	Chem. Commun. 2014, 50, 1731-1734.
**MoS_2/ Eosin-Y/graphene-oxide**	Triethanolamine	300 W Xenon lamp, λ > 420 nm	4.43	Angew. Chem. Int. Ed. 2013, 52, 13057-13061.

Supplementary Table 3. Comparison of photocatalytic hydrogen evolution activities of Janus $MoS_2/Bi_{12}O_{17}Cl_2$ bilayer with some representative bismuth-oxyhalide-based photocatalysts.

Photocatalyst	Hole scavenger	Light Source	PHE rate ($mmol \cdot h^{-1} \cdot g^{-1}$)	Reference
**Janus $MoS_2/Bi_{12}O_{17}Cl_2$ bilayer**	**Ascorbic acid**	**300 W Xenon lamp, λ > 420 nm**	**33**	**This work**
**Carbon-doped BiOCl/NiO_x**	Triethanolamine	300 W Xenon lamp, full spectrum	0.42	Adv. Funct. Mater. 2015, 25, 2189-2201.
**Black-BiOCl-nanosheets/Pt**	Triethanolamine	300 W Xenon lamp, λ > 420 nm	$7.92 \cdot 10^{-2}$	J. Power Sources 2015, 293, 409-415.
**Ultrathin-BiOCl**	No	500 W Xenon lamp, full spectrum	$3.5 \cdot 10^{-2}$	Chem. Eur. J. 2015, 21, 1-7.
**BiOCl/Au/MnO_x**	No	500 W Xenon lamp, full spectrum	$1.07 \cdot 10^{-2}$	Appl. Catal. B 2015, 162, 470-474.
**BiOCl/copper-phthalocyanine/rhodamine-B**	Methanol	500 W Xenon lamp, full spectrum	0.4	Appl. Catal. B 2013, 132, 315-320.
**$Bi_{24}O_{31}Br_{10}$**	Methanol	300 W Xenon lamp, λ > 400 nm	$6.7 \cdot 10^{-2}$	ACS Catal. 2014, 4, 954-961.

Supplementary Table 4. Comparison of photocatalytic hydrogen evolution activities of Janus $MoS_2/Bi_{12}O_{17}Cl_2$ bilayer with some representative ultrathin photocatalysts.

Photocatalyst	Hole scavenger	Light Source	PHE rate ($mmol \cdot h^{-1} \cdot g^{-1}$)	Reference
**Janus $MoS_2/Bi_{12}O_{17}Cl_2$ bilayer**	**Ascorbic acid**	**300 W Xenon lamp, λ > 420 nm**	**33**	**This work**
**Liquid-exfoliated C_3N_4 + Pt**	Triethanolamine	300 W Xenon lamp, λ > 420 nm	1.86	Adv. Mater. 2013, 25, 2452-2456.
**C_3N_4 nanosheets by thermal oxidation etching + Pt**	Triethanolamine	300 W Xenon lamp, λ > 420 nm	0.65	Adv. Funct. Mater. 2012, 22, 4763-4770.
**$Pb_2Nb_3O_{10}$-nanosheets/Pt**	Methanol	300 W Xenon lamp, full spectrum	0.1	ChemCatChem 2015, 7, 584-587.
**$K_4Nb_6O_{17}$-nanosheets**	Methanol	300 W Xenon lamp, full spectrum	1.66	Small 2014, 14, 2820-2825.
**CdS-nanosheets**	Na_2SO_3	300 W Xenon lamp, λ > 420 nm	$4.11 \cdot 10^{-2}$	Chem. Commun. 2013, 49, 9803-9805.
**$HNbWO_6$-nanosheets/Pt**	Triethanolamine	300 W Xenon lamp, full spectrum	0.4	Chem. Commun. 2015, 51, 15125-15128.
**HNb_3O_8-nanosheets/Pt**	Triethanolamine	125 W high-pressure Hg lamp, full spectrum	0.6	J. Mater. Chem. A 2015, 3, 20627-20632.

Supplementary Note 1

Characterizations of bulk layered $Bi_{12}O_{17}Cl_2$ nanosheets (BOC). SEM image of BOC revealed the sheet-shaped structures with thicknesses of several nanometers and micrometer-sized widths and lengths (**Supplementary Fig. 1a**). HRTEM image (**Supplementary Fig. 1b**) taken from the in-plane region of BOC nanosheets revealed two sets of lattices perpendicular to each other with an equal

fringe spacing of 2.71 Å, which respectively corresponded to (200) and (020) planes of the tetragonal $Bi_{12}O_{17}Cl_2$ phase, indicating both of the bottom and top surfaces of BOC were {001} facets. The corresponding selected-area electron diffraction (SAED) pattern (**Supplementary Fig. 1c**) indicated the single-crystalline characteristic of BOC. The angle between the (020) and (200) planes labeled in SAED pattern was measured to be 45°, which was in agreement with the theoretical value. This observation indicated that the set of diffraction spots in SAED can be indexed as the [001] zone axis of the tetragonal $Bi_{12}O_{17}Cl_2$, further confirming that the as-synthesized BOC nanosheets were exposed with {001} facets on both the top and the bottom surfaces and two {200} facets and two {020} facets on the lateral surfaces (**Supplementary Fig. 1f**). Furthermore, the side-view HRTEM image of BOC nanosheets (**Supplementary Fig. 1d**) revealed that bulk layered $Bi_{12}O_{17}Cl_2$ consisted of many single-layered nanosheets with a thickness of ca. 0.71 nm (**Supplementary Fig. 1e**), which demonstrated that the thickness of $Bi_{12}O_{17}Cl_2$ monolayer along [001] direction was 0.71 nm, agreeing well with its theoretical value (**Fig. 1i** and **Supplementary Fig. 2**, and **Supplementary Note 2**)[1-4].

Characterizations of bulk layered MoS_2 nanosheets (MS). Bulk layered MoS_2 nanosheets were synthesized via minor modification of a previously reported hydrothermal route[5]. SEM image of MS revealed the sheet-shaped structures with thicknesses of several nanometers and in-plane size of several hundred nanometers (**Supplementary Fig. 1g**). HRTEM image (**Supplementary Fig. 1h**) taken from the in-plane region of MS nanosheets showed distinct lattice fringes of 2.75 Å with 60° angles attributed to the (100) and (010) planes of 2H-MoS_2, respectively, indicating both of the bottom and top surfaces of MS were {001} facets. The corresponding selected-area electron

diffraction (SAED) pattern (**Supplementary Fig. 1i**) indicated the single-crystalline characteristic of MS. Meanwhile, the interplane angle of 60° of the two planes of (010) and (100) in the fast Fourier transform pattern as well as the well-defined hexagonal symmetry without additional points matched well with those calculated from hexagonal crystal graphic parameters of 2H-MoS_2, further confirming that the as-synthesized MS nanosheets were exposed with {001} facets on both the top and the bottom surfaces (**Supplementary Fig. 1l**). Furthermore, the side-view HRTEM image of MS nanosheets (**Supplementary Fig. 1j**) showed the lattice fringes spacing of 0.68 nm (**Supplementary Fig. 1k**), which was assigned to that of (001) planes of MoS_2, demonstrating the thickness of MoS_2 monolayer along [001] direction was 0.68 nm.

Supplementary Note 2

Similar with the reported Bi_2WO_6[13], which had a c = 16.427 Å, a unit cell consisting of two Bi_2WO_6 monolayers, but a monolayer thickness of 0.5c = 0.8 nm, $Bi_{12}O_{17}Cl_2$ had a c = 35.2 Å and a unit cell consisting of five $Bi_{12}O_{17}Cl_2$ monolayers (**Supplementary Fig. 2a**). In its each unit cell, the arrangements of the constituent five $Bi_{12}O_{17}Cl_2$ monolayers are along c axis. The Cl atoms in each $Bi_{12}O_{17}Cl_2$ monolayers are not in the same line along c axis, resulting in that each $Bi_{12}O_{17}Cl_2$ unit cell consists of five $Bi_{12}O_{17}Cl_2$ monolayers. These theoretical crystal structures were directly evidenced by the side-view atomic-resolution HAADF-STEM image and the corresponding EELS elemental maps of bulk layered $Bi_{12}O_{17}Cl_2$ nanosheets (**Supplementary Fig. 2c-2f**). During the lithium intercalation based liquid exfoliation, bulk layered $Bi_{12}O_{17}Cl_2$ would be exfoliated into $Bi_{12}O_{17}Cl_2$ monolayers, but not $Bi_{12}O_{17}Cl_2$ unit cells, because both $Bi_{12}O_{17}Cl_2$ monolayers in each

$Bi_{12}O_{17}Cl_2$ unit cell and $Bi_{12}O_{17}Cl_2$ unit cells in bulk layered $Bi_{12}O_{17}Cl_2$ were assembled by van der Waals forces, while a $Bi_{12}O_{17}Cl_2$ monolayer was a smaller unit than a $Bi_{12}O_{17}Cl_2$ unit cell (**Supplementary Fig. 2b**). As $Bi_{12}O_{17}Cl_2$ has a c = 35.2 Å and its each unit cell consisted of five $Bi_{12}O_{17}Cl_2$ monolayers, we undoubtedly conclude that the thickness of $Bi_{12}O_{17}Cl_2$ monolayer should be 0.2c = 0.704 nm. This theoretical value was well consistent with the experimental values measured from the AFM images, side-view TEM images, and side-view atomic-resolution HAADF-STEM image (**Supplementary Fig. 2g-2m**).

Supplementary Note 3

Confirmation of the single-layered structure of 1L-BOC. Organolithium chemistry was used to exfoliate layered $Bi_{12}O_{17}Cl_2$ nanosheets into their single-layered counterparts[6]. XRD patterns (**Supplementary Fig. 3a**) of BOC and 1L-BOC revealed that both of their peaks could be well indexed to the tetragonal $Bi_{12}O_{17}Cl_2$ phase (JCPDS NO 37-702) with lattice constant of a = b = 5.443 Å, and c = 35.20 Å (each unit cell consists of five $Bi_{12}O_{17}Cl_2$ monolayers) and that no peaks of any other phases were detected, suggesting that the as-prepared BOC and 1L-BOC were high-purity $Bi_{12}O_{17}Cl_2$ single-crystals. 1L-BOC showed much broader XRD peaks than BOC, indicating the successful exfoliation of bulk layered $Bi_{12}O_{17}Cl_2$ nanosheets by organolithium chemistry. TEM image (**Supplementary Fig. 3b**) of 1L-BOC revealed micrometer-sized nanosheets. Also, the nearly transparent feature of the nanosheets indicated their single-layered structures. 3-D topographic atomic force microscopy (AFM) image (**Supplementary Fig. 3c**) and its corresponding height profiles (**Supplementary Fig. 3d**) revealed that the as-prepared 1L-BOC had an average thickness

of ca. 0.71 nm, which agreed well with the theoretical thickness of $Bi_{12}O_{17}Cl_2$ monolayer along the [001] direction (**Fig. 1i** and **Supplementary Fig. 2**, and **Supplementary Note 2**), providing direct and solid evidence for the formation of $Bi_{12}O_{17}Cl_2$ monolayers. HRTEM image (**Supplementary Fig. 3e**) taken from the in-plane region of 1L-BOC revealed two sets of lattices perpendicular to each other with an equal fringe spacing of 2.71 Å, which respectively corresponded to (200) and (020) planes of the tetragonal $Bi_{12}O_{17}Cl_2$ phase, indicating both of the bottom and top surfaces of BOC were {001} facets. The preferential [001] orientation of 1L-BOC indicated that its synthesis was realized by exfoliating BOC along the Z-axis.

Confirmation of the asymmetric structure of 1L-BOC composed of only [Cl_2] layers and oxygen-deficient [$Bi_{12}O_{17}$] layers. The cross-sectional atomic structures of 1L-BOC were imaged directly by the aberration-corrected high-angular annular dark field scanning TEM (HAADF-STEM). It is known that the signal intensity of atoms shown in HAADF-STEM image is approximately proportional to Z^2, where Z is the atomic number. As the atomic numbers of Bi, Cl, and O are 83, 17, and 8, respectively, it could be inferred that, in **Supplementary Fig. 3f**, the brightest spots on the second-topmost layers, the second brightest spots on the bottommost layers, and the darkest spots on the second-bottommost and topmost layers were ascribed to Bi, Cl, and O, respectively. This observation also indicated that 1L-BOC had an asymmetric structure composed of only [Cl_2] layers and [$Bi_{12}O_{17}$] layers.

Confirmation of the presence of oxygen vacancies (OVs) on the surface of 1L-BOC. Low temperature electron paramagnetic resonance (EPR) spectra (**Supplementary Fig. 3g**) of 1L-BOC showed a typical signal peak of oxygen vacancies (OVs) at g = 2.003, but absent in BOC, indicating

the presence of oxygen vacancies on the 1L-BOC. These OVs were further proven to be confined only on the surface of 1L-BOC, as evidenced by a shoulder-like light absorption bands at longer wavelength without changing the intrinsic optical absorption edge shown in DRS spectra (**Supplementary Fig. 3h**). The BET surface area (**Supplementary Fig. 3i**) of the 1L-BOC was about 40.91 $m^2 g^{-1}$, 4.95 times larger than that of BOC (8.26 $m^2 g^{-1}$).

Supplementary Note 4

Confirmation of the single-layered structure of 1L-MS. Organolithium chemistry was used to exfoliate layered MoS_2 nanosheets into their single-layered counterparts[6]. XRD patterns (**Supplementary Fig. 4a**) of MS and 1L-MS revealed that both of their peaks could be well indexed to the hexagonal MoS_2 phase (JCPDS NO 6-97) and that no peaks of any other phases were detected, suggestting that the as-prepared MS and 1L-MS were high-purity MoS_2 single-crystallines. 1L-MS showed much broader XRD peaks than MS, indicating the successful exfoliation of bulk layered MoS_2 nanosheets by organolithium chemistry to craft MoS_2 monolayers. The exclusive presence of the (00a) (a = 1, 2, 4, 6, 8) diffraction peaks in 1L-MS revealed its highly preferred (001) orientation. TEM image (**Supplementary Fig. 4b**) of 1L-MS revealed nanosheets with in-plane size of several hundred nanometers, and thier nearly transparent feature indicated the single-layered structures. 3-D topographic AFM image (**Supplementary Fig. 4c**) and its corresponding height profiles (**Supplementary Fig. 4d**) revealed that the as-prepared 1L-MS had an average thickness of ca. 0.68 nm, which agreed well with the thickness of single-unit-cell MoS_2 slab along the [001] direction, providing direct and solid evidence for the formation of MoS_2 monolayers.

Confirmation of the presence of the metallic phase in 1L-MS. Supplementary Fig. 4e showed the temperature dependent electrical resistivity of MS and 1L-MS. When the temperature arose, the electrical resistivity of MS decreased, indicating the semiconductor characteristic, while that of 1L-MS increased, suggesting the metallic characteristic. The metallic characteristic of 1L-MS was also evidenced by its much lower electrical resistivity than that of MS, because the metallic phase could effectively promote the electron mobility. Unlike MS with Mo 3d XPS peaks locating only on 229.46 and 232.43 eV, corresponding to 2H semiconducting phase of MoS_2, 1L-MS revealed the Mo 3d XPS peaks of both 2H semiconducting phase and 1T metallic phase (228.82 and 231.82 eV) of MoS_2 (**Supplementary Fig. 4f**). The 1T metallic phase concentration in 1L-MS calculated by the ratio of 1T to 2H peak areas was estimated to be around 82%, matching well with the values reported previously in Li-exfoliated MoS_2 monolayers. The side-view HAADF-STEM image of 1L-MS revealed that the lateral atoms were orderly arranged according to the 1T metallic phase of MoS_2 (**Supplementary Fig. 4g-4h**).

Supplementary Note 5

The chemical compositions of the Janus bilayer junctions: As shown in **Supplementary Fig. 5**, XRD patterns of BOC-MS and BOC-MS-A1 displayed the XRD peaks of both 1L-BOC and 1L-MS, indicating the successful assembly of 1L-MS on 1L-BOC by OV chemistry.

Supplementary Note 6

Tuning the surface OV concentrations of $Bi_{12}O_{17}Cl_2$ monolayers by aerobic calcination. The relative atomic concentrations can be estimated by normalizing XPS peak area (**Supplementary Fig. 6**) with atomic sensitivity factor according to the following equation: $n_1/n_2 = (I_1/S_1)/(I_2/S_2)$, where n_1 and n_2 represent the atomic numbers of element **1** and **2**, I_1 and I_2 are the XPS peak area, and S_1 and S_2 are the sensitivity factors. The calculated OV concentrations were 11%, 5.3% and 0, for 1L-BOC, 1L-BOC-1 and 1L-BOC-2, respectively.

Supplementary Note 7

Identification of OVs on $Bi_{12}O_{17}Cl_2$ monolayers as the driving force to assemble $Bi_{12}O_{17}Cl_2$ and MoS_2 monolayers. To check whether this oriented assembly was initiated by OV chemistry, we then investigated the assembling behavior of MoS_2 monolayers and $Bi_{12}O_{17}Cl_2$ monolayers with different amounts of OVs. Aerobic calcination resulted in OV decrease in $Bi_{12}O_{17}Cl_2$ monolayers (**Supplementary Fig. 6** and **Supplementary Table 1**), as verified by calcination-time dependent decay of electron paramagnetic resonance peak at g = 2.001 (**Supplementary Fig. 7a**). **Supplementary Fig. 7b-7i** showed that the amount of MoS_2 loaded on $Bi_{12}O_{17}Cl_2$ decreased with OV quenching, and that the assembling behavior annihilated when OV was totally perished. To quantitatively express this relationship, we plotted the surface coverage proportions of MoS_2 on $Bi_{12}O_{17}Cl_2$ versus OV concentration as the **Supplementary Fig. 7j**. Then, a splendid linearity relationship was observed, indicating the strong dependence of assembly ability on OV amounts.

This provided direct evidence that OV chemistry imparted by $Bi_{12}O_{17}Cl_2$ monolayers was the driving force to assemble monolayers of MoS_2 and $Bi_{12}O_{17}Cl_2$.

Supplementary Note 8

Stability of the Janus bilayer structure during long-term photocatalytic tests. Even after 100 h (10 times of successive cycles) of photocatalytic experiments, BOC-MS still almost completely retained its morphology and chemical composition, verifying the excellent robustness of its Janus bilayer structure.

Supplementary Note 9

We provided the magnified image of the **Fig. 1m** and the two additional side-view TEM images of BOC-MS as **Supplementary Fig. 19a-19c**. All these three TEM images revealed no gap between MoS_2 and $Bi_{12}O_{17}Cl_2$ monolayers in the Janus bilayers. These observations also demonstrated a very short interfacial distance between MoS_2 and $Bi_{12}O_{17}Cl_2$ monolayers, which well agreed with the results of the side-view HAADF-STEM image shown in **Fig. 1n**, and indicated that the formation of the interfacial Bi-S bonds was spatially favorable. Besides XPS (**Fig. 3q**) and EXAFS (**Fig. 3r**) spectra, the Raman spectra, diffuse reflectance Fourier transform infrared (FTIR) curves, and thermogravimetric and differential thermal analysis (TG-DTA) were carried out to provide the new evidences to further support the existence of the interfacial Bi-S bonds between MoS_2 and $Bi_{12}O_{17}Cl_2$ monolayers in our designed 2-D Janus bilayer junctions. We observed the Raman

(**Supplementary Fig. 19e**) and FTIR (**Supplementary Fig. 19f**) signals of the Bi-S bonds, only in the Janus bilayer junctions (BOC-MS and BOC-MS-A1), but not in the physical mixtures (BOC-MS-Mix) of 1L-MS and 1L-BOC. Moreover, the intensities of these two signals increased with increasing the Bi-S bond concentration. Furthermore, because of the interfacial chemical bonding via the Bi-S bonds, MoS_2 and $Bi_{12}O_{17}Cl_2$ monolayers in the Janus bilayers could enhance their respective thermal decomposition temperatures by 104 and 87 ^oC (**Supplementary Fig. 19g-19j**). In contrast, no obvious improvement was observed in the thermal decomposition temperature of BOC-MS-Mix without the interfacial chemical Bi-S bonds (**Supplementary Fig. 19i**). Moreover, the excellent linear relationship between the Bi-S bond concentration and the photocurrent density (**Supplementary Fig. 19d** and **Supplementary Fig. 19k**) highlighted the crucial role of the Bi-S bonds in directing the charge flow from $Bi_{12}O_{17}Cl_2$ to MoS_2 monolayers in the Janus bilayers, and thus could act as an indirect evidence for the interfacial Bi-S bonds. Therefore, all these characterizations provided further strong evidences for the existence of Bi-S bonds in the interface between $Bi_{12}O_{17}Cl_2$ and MoS_2 monolayers.

Supplementary Note 10

To further determine the photo-deposition sites of MnO_x, we carried out the side-view TEM image of the Janus bilayer junctions photo-deposited with MnO_x. As shown in **Supplementary Fig. 22**, the TEM image directly evidenced that all the MnO_x nanoparticles were deposited on the [Cl_2] end-faces of $Bi_{12}O_{17}Cl_2$ monolayers, not on MoS_2 monolayers, further testifying our proposed charge

flow pathways that the photogenerated holes were directionally separated by the internal electric field to [Cl_2] end-faces of $Bi_{12}O_{17}Cl_2$ monolayers.

Supplementary Note 11

The HER polarization curves shown in **Supplementary Fig. 25** revealed that the electrocatalytic HER activity of BOC-MS was excellent with a low onset overpotentials of 0.11 V and a small Tafel slope of 52 mV decade^{-1}. By means of this remarkable hydrogen-evolving catalysis behavior, BOC-MS, despite being noble-metal-free, still delivered a superior PHE activity. 1L-BOC, despite having abundant coordinately unsaturated Bi atoms, still displayed an electrocatalytic HER activity lower than MS and far lower than 1L-MS. These comparison indicated that, for BOC-MS, the hydrogen-evolving catalysis proceeded mainly on 1L-MS, rather on 1L-BOC.

Supplementary Methods

Electrochemical Measurements: The indium doped tin oxide (ITO, China Southern Glass Co., Ltd., Shenzhen, China) substrates were first ultrasonically cleaned in distilled water, absolute ethanol, and isopropanol for 15 min sequentially. Both edges of the conducting glass substrates were then covered with adhesive tape. Typically, the aqueous slurries of the samples were spread on an ITO glass substrate with a glass rod, using adhesive tapes as spaces. The suspension was prepared by grinding 20 mg of samples, 40 μL of PEDOT-PSS (Sigma-Aldrich, 1.3-1.7%) aqueous solution, and 200 μL of water. The resulting film was dried in air and annealed at 150 ℃ for 10 min, yielding an

electrode with catalyst loading amount of ca. 0.254 mg cm^{-2}. The photocurrents were measured by an electrochemical analyzer (CHI660D, Shanghai, China) in a standard three-electrode system with the samples as the working electrodes, a Pt foil as the counter electrode, and a saturated calomel electrode (SCE) as a reference electrode. A 300 W Xe arc lamp equipped with a band-pass filter (420 nm) was utilized as a light source.

Electrocatalytic H_2 Generation was conducted on CHI660D Instruments in a standard three-electrode system with catalysts loaded on glass carbon electrode (5 mm in diameter) as the working electrode, coiled platinum wire and graphite rod as the counter electrode, Ag/AgCl electrode as the reference electrode and 0.5 M H_2SO_4 (sparged with pure H_2) as the electrolyte. All the potentials were calibrated against and converted to a reversible hydrogen electrode (RHE) following the equation: $E_{RHE} = E_{SCE} + 0.213$ V. Linear sweep voltammetry (LSV) was carried out at 5 mV s^{-1} for the polarization curves and 0.1 mV s^{-1} for Tafel plots.

Calculation of PHE quantum yields. The quantum yield of PHE over the Janus bilayer junctions was measured under the same photocatalytic reaction condition as that shown in **Figure 2h**. The 300 W Xe arc lamp equipped with a band-pass filter (320, 340, 360, 380, 400, 420, 450, 470, 500, 550, 600, and 700 nm) was used as the light source. The quantum yield (η) was determined by a well-established equation: η (%) = (number of reacted electrons)/(number of incident photons) × 100% = (number of evolved H_2 molecules ×2)/(number of incident photons) × 100%. The number of incident photons (N) was calculated according to the equation: $N = (E\lambda)/(hc)$, where E, λ, h, and c are the energy of incident photons, the wavelength of the incident monochromatic light, the Planck

constant, and the light speed, respectively. The intensity of incident monochromatic light was measured by a calibrated Si photodiode (HAMAMATSU S2281) and the irradiation area was ca. 6.23 cm^2. The measured intensities of the incident monochromatic light with wavelengths at 320, 340, 360, 380, 400, 420, 450, 470, 500, 550, 600, and 700 nm, which were shown in **Supplementary Fig. 11**, were almost the same as the reported value[12].

IEF magnitude calculation. We calculated the variation of IEF magnitude of the pristine and C-doped Bi_3O_4Cl nanosheets by using the following equation developed by Kanata et al[7-9]:

$$F_S = (-2V_S \rho / \varepsilon\varepsilon_0)^{1/2} \qquad (1)$$

Where *Fs* is the IEF magnitude, *Vs* is the surface voltage, ρ is the surface charge density, ε is the low-frequency dielectric constant, and ε_0 is the permittivity of free space. The above equation reveals that the IEF magnitude is mainly determined by the surface voltage and the charge density because ε and ε_0 are two constants. In order to evaluate the IEF magnitude variation in bulk and single-layered $Bi_{12}O_{17}Cl_2$, we carefully figured out their surface voltages by open-circuit potentials measurements and charge densities by the simultaneous potentiometric and conductimetric titrations method.

Surface charge density of bulk and single-layered $Bi_{12}O_{17}Cl_2$ as function of pH was calculated by applying the following equation and using K_1 and K_2 values obtained from the simultaneous potentiometric and conductimetric titrations. The equilibrium constants K_1 and K_2 can be experimentally determined by the application of Henderson–Hasselbach equation in the

simultaneous potentiometric and conductimetric titration data. From equilibrium constants, the surface charge density of Bi_3O_4Cl nanosheets as function of pH values can be calculated by the following equation:

$$\rho_0 = (\frac{F}{A})[(\frac{10^{-2pH} - K_1K_2}{10^{-2pH} + 10^{-2pH}K_1 + K_1K_2})N_T] \qquad (2)$$

Where *F* is the Faraday constant, *A* is the total surface area, *NT* is the total number of moles of surface sites, and K_1 and K_2 are the acid equilibrium constants.

Supplementary References:

1. Xiao, X. Y., Jiang, J. & Zhang, L. Z. Selective oxidation of benzyl alcohol into benzaldehyde over semiconductors under visible light: the case of $Bi_{12}O_{17}Cl_2$ nanobelts. *Appl. Catal. B* **142**, 487-493 (2013).

2. Deng, H., Wang, J. W., Peng, Q., Wang, X. & Li, Y. D. Controlled hydrothermal synthesis of bismuth oxyhalide nanobelts and nanotubes. *Chem. Eur. J.* **11**, 6519 –6524 (2005).

3. Chen, X. Y., Huh, H. S. & Lee, S. W. Controlled synthesis of bismuth oxo nanoscale crystals (BiOCl, $Bi_{12}O_{17}Cl_2$, α-Bi_2O_3, and $(BiO)_2CO_3$) by solution-phase methods. *J. Solid State Chem.* **180**, 2510-2516 (2007).

4. Xiao, X. et al. Oxygen-rich bismuth oxyhalides: generalized one-pot synthesis, band structures and visible-light photocatalytic properties. *J. Mater. Chem.* **22**, 22840-22843 (2012).

5. Xie, J. F. et al. Defect-rich MoS_2 ultrathin nanosheets with additional active edge sites for enhanced electrocatalytic hydrogen evolution. *Adv. Mater*. **25**, 5807–5813 (2013).

6. Ramakrishna Matte, H. S. S. et al. MoS_2 and WS_2 analogues of graphene. *Angew. Chem. Int. Ed*. **49**, 4059 –4062 (2010).

7. Lefebvre, P., Allegre, J., Gil, B. & Mathieu, H. Time-resolved photoluminescence as a probe of internal electric fields in GaN-(GaAl)N quantum wells. *Phys. Rev. B* **59**, 15363-15367 (1999).

8. Morello, G. et al. Intrinsic optical nonlinearity in colloidal seeded grown CdSe/CdS nanostructures: photoinduced screening of the internal electric field. *Phys. Rev. B* **78**, 195313 (2008).

9. Im, J. S. et al. Reduction of oscillator strength due to piezoelectric fields in GaN/$Al_xGa_{1-x}N$ quantum wells. *Phys. Rev. B* **57**, R9435- R9438 (1998).

10. Jiang, J., Zhang, X., Sun, P. B. & Zhang, L. Z. ZnO/BiOI heterostructures: photoinduced charge-transfer property and enhanced visible-light photocatalytic activity. *J. Phys. Chem. C* **115**, 20555–20564 (2011).

11. Zhang, X., Zhang, L. Z., Xie, T. F. & Wang, D. J. Low-temperature synthesis and high visible-light-induced photocatalytic activity of BiOI/TiO_2 heterostructures *J. Phys. Chem. C* **113**, 7371–7378 (2009).

12. Yi, Z. G. et al. An orthophosphate semiconductor with photooxidation properties under visible-light irradiation. *Nat. Mater*. **9**, 559–564 (2010).

13. Zhou, Y. G. et al. Monolayered Bi_2WO_6 nanosheets mimicking heterojunction interface with open surfaces for photocatalysis. *Nat. Commun*. **6**, 8340 (2015).
